# *Ehmt2* inactivation in pancreatic epithelial cells shapes the transcriptional landscape and inflammation response of the whole pancreas

**DOI:** 10.3389/fgene.2024.1412767

**Published:** 2024-06-14

**Authors:** Gareth Pollin, Angela J. Mathison, Thiago M. de Assuncao, Anju Thomas, Atefeh Zeighami, Ann Salmonson, Hongfei Liu, Guillermo Urrutia, Pallavi Vankayala, Stephen J. Pandol, Johnny C. Hong, Michael T. Zimmermann, Juan Iovanna, Victor X. Jin, Raul Urrutia, Gwen Lomberk

**Affiliations:** ^1^ Linda T. and John A. Mellowes Center for Genomic Sciences and Precision Medicine, Medical College of Wisconsin, Milwaukee, WI, United States; ^2^ Division of Research, Department of Surgery, Medical College of Wisconsin, Milwaukee, WI, United States; ^3^ Department of Medicine, Cedars-Sinai Medical Center, Los Angeles, CA, United States; ^4^ Division of Transplantation, Department of Surgery, College of Medicine, Pennsylvania State University, Hershey, PA, United States; ^5^ Department of Biochemistry, Medical College of Wisconsin, Milwaukee, WI, United States; ^6^ Clinical and Translational Sciences Institute, Medical College of Wisconsin, Milwaukee, WI, United States; ^7^ Centre de Recherche en Cancérologie de Marseille (CRCM), Institut National de la Santé et de la Recherche médicale (INSERM) U1068, CNRS UMR 7258, Parc Scientifique et Technologique de Luminy, Aix-Marseille Université and Institut Paoli-Calmettes, Marseille, France; ^8^ Division of Biostatistics, Institute for Health and Equity, Medical College of Wisconsin, Milwaukee, WI, United States; ^9^ Department of Pharmacology and Toxicology, Medical College of Wisconsin, Milwaukee, WI, United States

**Keywords:** EHMT2 (G9a), acute pancreatitis, inflammation, epigenetics, gene expression, RNA-seq, spatial transcriptomics, conditional knock out mice

## Abstract

**Introduction:** The Euchromatic Histone Methyl Transferase Protein 2 (EHMT2), also known as G9a, deposits transcriptionally repressive chromatin marks that play pivotal roles in the maturation and homeostasis of multiple organs. Recently, we have shown that *Ehmt2* inactivation in the mouse pancreas alters growth and immune gene expression networks, antagonizing Kras-mediated pancreatic cancer initiation and promotion. Here, we elucidate the essential role of Ehmt2 in maintaining a transcriptional landscape that protects organs from inflammation.

**Methods:** Comparative RNA-seq studies between normal postnatal and young adult pancreatic tissue from *Ehmt2* conditional knockout animals (*Ehmt2* ^*fl/fl*^) targeted to the exocrine pancreatic epithelial cells (*Pdx1-Cre* and *P48* ^*Cre/+*^), reveal alterations in gene expression networks in the whole organ related to injury-inflammation-repair, suggesting an increased predisposition to damage. Thus, we induced an inflammation repair response in the *Ehmt2* ^*fl/fl*^ pancreas and used a data science-based approach to integrate RNA-seq-derived pathways and networks, deconvolution digital cytology, and spatial transcriptomics. We also analyzed the tissue response to damage at the morphological, biochemical, and molecular pathology levels.

**Results and discussion:** The *Ehmt2* ^*fl/fl*^ pancreas displays an enhanced injury-inflammation-repair response, offering insights into fundamental molecular and cellular mechanisms involved in this process. More importantly, these data show that conditional *Ehmt2* inactivation in exocrine cells reprograms the local environment to recruit mesenchymal and immunological cells needed to mount an increased inflammatory response. Mechanistically, this response is an enhanced injury-inflammation-repair reaction with a small contribution of specific Ehmt2-regulated transcripts. Thus, this new knowledge extends the mechanisms underlying the role of the Ehmt2-mediated pathway in suppressing pancreatic cancer initiation and modulating inflammatory pancreatic diseases.

## 1 Introduction

The pancreas arises from a small cluster of cells in the embryonic gut tube during development (Joglekar et al., 2007). This process is tightly regulated by a complex network of signaling pathways and transcription factors, which control pancreatic progenitor cell specification, proliferation, and differentiation (Jarc et al., 2023). This glandular organ comprises different cell types, including endocrine cells that produce hormones and exocrine cells that produce digestive enzymes (Overton and Mastracci, 2022). The proper development and function of the pancreatic cells are critical for maintaining health and preventing diseases, such as diabetes and pancreatitis (Karpińska and Czauderna, 2022). These conditions are associated with defects in the molecular pathways that regulate pancreas development and function (Thrower et al., 2008; Polireddy and Chen, 2016). However, there is limited knowledge on the precise mechanisms underlying these defects, and further research is still needed to better understand the complex molecular interactions within the pancreas.

Acute pancreatitis has a rising global incidence and is associated with significant morbidity and mortality. In severe cases, mortality rates can reach 30%–40% (Iannuzzi et al., 2022). The pathogenesis of acute pancreatitis is multifaceted and involves both local and systemic inflammatory responses. Epigenetic regulatory mechanisms play an important role in controlling the inflammatory cascade (Zhou et al., 2022). Various studies have investigated the role of epigenetic modifications in regulating inflammation and acute pancreatitis (Sandoval et al., 2016; Sun et al., 2021). DNA methylation is one of the most extensively studied epigenetic modifications in the context of acute pancreatitis. Studies have shown altered DNA methylation levels on genes involved in inflammation and oxidative stress in the pancreas during acute pancreatitis episodes (Natale et al., 2019; Sun et al., 2021). For instance, promoter hypermethylation of anti-inflammatory genes such as *IL10* and *SOCS3* has been associated with decreased expression and increased inflammation in the pancreas (Yin et al., 2015). Similarly, hypomethylation of pro-inflammatory *NFKB1* promoter regions increases expression and enhances pancreas inflammation. Histone modifications have also been implicated in the pathogenesis of acute pancreatitis. For example, histone H4 acetylation in the IL-1β promoter region correlates with increased expression of this pro-inflammatory cytokine in the pancreas (Pedersen et al., 2022). However, further research is needed to fully understand the complex interplay between epigenetic regulators and the inflammatory cascade in acute pancreatitis, which could lead to the development of new therapies for this debilitating disease.

Euchromatic histone-lysine N-methyltransferase 2 (EHMT2/G9a) is a methyltransferase that catalyzes the mono- and dimethylation of lysine nine on histone H3 (H3K9me1/2), leading to transcriptional repression. EHMT2 has been implicated in regulating various cellular processes beyond gene expression, including cellular differentiation and DNA repair (Jan et al., 2021). While EHMT2 directly regulates the differentiation and function of various immune cell types, including T cells, B cells, and macrophages, several studies also support the role of EHMT2 in non-immune cells to control inflammatory responses (Scheer and Zaph, 2017; Mourits et al., 2021). For instance, EHMT2 represses the expression of pro-inflammatory cytokines, such as TNF, in tumor cells to promote breast cancer recurrence (Mabe et al., 2020). Studies on vascular smooth muscle cells have also implicated EHMT2 in attenuating the IL-6 inflammatory response in atherosclerotic lesions (Harman et al., 2019). Furthermore, liver-specific *Ehmt2* knockout leads to an enhanced proinflammatory response in lipopolysaccharide (LPS)-induced liver injury model (Lu et al., 2019). Recently, we have shown that *Ehmt2* inactivation antagonizes oncogenic Kras-mediated pancreatic cancer initiation and promotion by altering growth and immune gene expression networks (Urrutia et al., 2021). *Ehmt2* knockout driven by either *Pdx1-Cre* or *P48* ^*Cre/+*^ demonstrated that this pathway is not required for pancreas exocrine development and is tolerated in this organ under basal contexts. However, the impact of Ehmt2 on the transcriptome during early development and under the inflammatory stressor of acute pancreatitis remains unknown.

Here, we investigate the role of Ehmt2 in postnatal murine pancreas development and caerulein-induced acute pancreatitis. *Ehmt2* inactivation results in distinct transcriptional landscapes during pancreatic maturation, suggesting an increased susceptibility of this organ to an injury-inflammation-repair process. Congruently, in response to the induction of acute pancreatitis, we show that *Ehmt2* inactivation leads to a more aggressive inflammatory response. Thus, this study advances our understanding of the epigenetic regulation and molecular mechanisms that play a role in pancreatic development, homeostasis, and diseases.

## 2 Materials and methods

### 2.1 Mouse models and acute pancreatitis induction

Animal care and all experimental protocols were reviewed and approved by the Institutional Animal Care and Use Committees of Mayo Clinic Rochester (IACUC protocols A00002240-16 and A24815) and the Medical College of Wisconsin (AUA00005963). Mice were maintained in standard housing with controlled temperature, humidity, and light cycles and given standard rodent chow and water *ad libitum*. Tissues were collected and preserved in formaldehyde for a minimum of 24 h prior to transfer to 70% (v/v) ethanol for histological processing and examination. *B6.FVB-Tg(Pdx1-Cre)6Tuv/J* (*Pdx1-Cre*, IMSR Cat# JAX:014647, RRID: IMSR_JAX:014647) (Hingorani et al., 2003) and *Ptf1a* ^*TM*^ ^*1(cre)Hnak*^ */RschJ* (*P48* ^*Cre/+*^, IMSR Cat# JAX:023329, RRID: IMSR_JAX:023329) (Nakhai et al., 2007) were originally purchased from Jackson Laboratories. *Ehmt2 flox/flox* (*Ehmt2* ^*fl/fl*^) animals were generously provided by Dr. Oltz (Tachibana et al., 2007). Animals were maintained on a C57Bl/6 background, and genotyping procedures to confirm *Pdx1-Cre*;*Ehmt2* ^*fl/fl*^ and *P48* ^*Cre/+*^;*Ehmt2* ^*fl/fl*^ crosses have been described previously (Urrutia et al., 2021). Both sexes were included in the experiments. Animals that were used in ontogeny studies with no additional treatments were sacrificed at 10 days (postnatal, PN) or 4 weeks (young adult, YA). For induction of acute pancreatitis, cohorts of 4-week-old mice were fasted for 12 h before the first caerulein injection in accordance with prior studies (Ding et al., 2003; Vasseur et al., 2004; Strobel et al., 2007; Shigekawa et al., 2012; Choi et al., 2015; He et al., 2024). Caerulein or saline control was administered via IP injection at a dose of 50 μg/kg once an hour for a total of eight injections. Food was returned after the first dose. Animals were euthanized 18 h after the initial dose. Tissue was taken (*n* = 2-4) for RNA and histological analysis. Mice were euthanized using CO_2_ in accordance with institutional guidelines.

### 2.2 Serum analysis

Blood was collected from the animals by orbital puncture and serum was isolated for studying serum chemistry. Serum was mixed 1:1 with saline and their profile consisting of Glucose, Albumin, Globulin, Total Protein, Alanine Amino Transferase, Total bilirubin, Minerals (Sodium, calcium, Phosphorus) Blood Urea Nitrogen (BUN), and Alkaline phosphatase were measured using VetScan VS2 (Mathison et al., 2013).

### 2.3 Histological analysis

Pancreatic tissues were paraffin-embedded for sectioning, and sections were stained with hematoxylin and eosin (H&E) for pathological evaluation. Pancreatitis severity was assessed by assessing pancreatic tissue edema, inflammatory cell infiltration, and necrosis. The scoring system was adapted from (Moreno et al., 2006). It included an assessment of pancreatic tissue edema (on a scale from a minimum of 0 for no edema to a maximum of three for separated and disrupted acini), inflammatory cell infiltrate (on a scale from a minimum of 0 for no infiltrate to a maximum of 3 with infiltrate in the parenchyma for >50% of the lobules), and necrosis (on a scale from a minimum of 0 for absence of necrosis to a maximum of 3 with diffuse parenchymal necrosis for >10% of the parenchyma). To quantify the severity of the parameters, four random fields (×20 objective) per slide, each containing at least 1,000 cells per field, were imaged and assessed by the three scales. Mean ± SD scores were calculated for each parameter.

### 2.4 TUNEL assay

Formalin-fixed pancreatic tissues were paraffin-embedded and sectioned (5 µm). TUNEL analysis was carried out using the ApopTag Peroxidase *in situ* cell apoptosis detection kit (Millipore, S7100) according to the manufacturer’s directions. Slides were developed with Nova Red (Vector Laboratories) and counterstained with Mayer hematoxylin. Five random fields (×20 objective) per section, containing at least 1,000 cells per field, were imaged and counted (Urrutia et al., 2020).

### 2.5 RNA extraction, RNA-seq, and bioinformatics analysis

Preparation of RNA from tissue was performed as previously described (Urrutia et al., 2021). To reduce degradation during storage at −80°C, RNaseOUT (Invitrogen, Cat# 10777019) added to final elution. The RNA samples were quantified by Qubit (Invitrogen), and their quality was assessed using the Fragment Analyzer (Agilent). The samples with RINs >6 and DV200 > 80% were selected for library preparation. The pancreas RNA was then sequenced using the Illumina TruSeq RNA v2 library preparation kit and the Illumina High Seq-2000. The sequencing reads were mapped to the mouse reference transcriptome Gencode vM23 (GRCm38. p6), and at least 24 million mapped read pairs were obtained per sample. The resulting reads were processed through the Mellowes Center workflow, which includes MapRseq3 (Kalari et al., 2014) and EdgeR (McCarthy et al., 2012). Differential gene expression was based on a false discovery rate (FDR) < 0.1 and an absolute fold change (FC) ≥ | 2.0|. Pathway analysis of DEGs was done using RITAN (Zimmermann et al., 2019) and the MSigDB hallmark gene set collection (Liberzon et al., 2015), while gene network and upstream regulatory analyses were performed using Ingenuity^®^ Pathway Analysis (IPA^®^; Qiagen). To quantify immune-cell fractions in the young-adult/postnatal bulk RNA-seq comparisons, we performed digital cytometry analysis with the quanTiSeq algorithms, which allowed intra-sample and inter-sample comparisons of only immune cell type fractions (Finotello et al., 2019). The quanTIseq method was applied on DEGs from through an R package called Immunedeconv allowing for identification of immune cell composition despite the low overall immune cell population (Sturm et al., 2019). Using the same R package for the bulk RNA-seq comparisons that involved caerulein injections, the MCP-Counter algorithm was used, which allowed for an expanded breakdown of the complete cell composition with robust quantification of the absolute abundance of eight immune and stromal cell populations (Becht et al., 2016).

### 2.6 Reverse transcription quantitative real-time PCR (RT-qPCR)

Total RNA (2 μg) was used as a template for cDNA synthesis, using the RT^2^ First Strand Kit (Qiagen) according to manufacturer’s protocol. RT^2^ SYBR Green qPCR Mastermix (Qiagen) was used with the following reaction conditions: denaturation at 95°C for 10  min; 45 cycles of 15 s at 95 °C, 60 s at 60°C. Reactions were carried out using the CFX96 Real Time System, and real-time PCR data was analyzed using the CFX Maestro software v2.3 (Bio-Rad). Primer sequences for each transcript are provided in Supplementary Table S6.

### 2.7 Spatial transcriptomics

FFPE Pancreatic tissue sections (*Ehmt2* ^*+/+*^ and *Ehmt2* ^*fl/fl*^) were deparaffinized followed by H&E staining and imaging using BZ-X800 (Keyence). Thereafter, tissue was destained, decrosslinked, and permeabilized for processing with the CytAssist and Visium Spatial Gene Expression Kits (10x Genomics; Pleasanton, CA, United States). Library quality metrics were confirmed by fragment analysis and Kapa qPCR before sequencing according to manufacturer recommendations on the NovaSeq 6,000 (Illumina, San Diego, CA, United States). A sequencing depth of approximately 250–400 million read-pairs per sample was obtained. Sample processing, library preparation, and sequencing for this project was completed by the Mellowes Center for Genomic Sciences and Precision Medicine Center at the Medical College of Wisconsin (RRID:SCR_022926). The original read quality was checked by FastQC and FastQ Screen. Alignment, tissue detection, fiducial detection, and barcode/UMI counting were performed by Spaceranger Count. For read alignment, reference transcriptome was mouse mm10-2020-A with mouse Visium Probe_Set_v1.0. Seurat was used for downstream spatial data analysis. First, spots with zero counts were filtered out, and normalization was performed using the SCTransform method. Subsequently, dimension reduction and clustering were performed with PCA and UMAP analysis. Spatially expressed genes for each cluster were found using the default Seurat method. Spatially variable genes were found using two Moran’s I methods, specifically the Seurat clustering and UMAP. Results were integrated to Loupe Browser for data visualization. Each cell type was assigned to its respective cluster through manual review of the expression of a comprehensive set of marker genes, as per outlined by Zhou et al. (2022), and the analysis of genes of interest involved utilizing the scale value, either in the form of Log normalized or Feature sum.

### 2.8 Analysis of publicly available Ehmt2 ChIP-seq data generated across many cell types

To confirm direct Ehmt2 target genes, we compared the results of RNA-Seq with several EHMT2 ChIP-Seq experiments, using the ChIP-Atlas interface (Zou et al., 2022). Briefly, RNA-seq values were correlated with the presence of peaks within a window of *−/+*10 kb from the transcription start site of the gene, using both the hg38 and mm10 reference genomes. The data was numerically harmonized with values transformed from continuous to digital because they originated from the different cell types, with the following identifiers (SRX number corresponds to the sample identifier): HEK293: SRX738354, SRX973412, SRX973413, SRX973414, SRX973415, SRX973428, SRX973429; A549: SRX3010248, SRX3010249; Hep G2: SRX10478057, SRX10478058; IMR-32: SRX16322836; K-562: SRX5457324, SRX5457325; LNCAP: SRX17413109, SRX17413110; MCF-7: SRX7030880, SRX7030881, SRX7030882, SRX7030883; RD/18: SRX5316603; RH-41: SRX4561210; Cardiomyocytes: SRX2497443, SRX2497444, SRX2499660, SRX2499661, SRX2499669, SRX2499670; EpiSC: DRX013321; and ES cells: SRX348394, SRX348395.

## 3 Results

### 3.1 Loss of *Ehmt2* increases the propensity of the normal pancreas to injury-inflammation

**FIGURE 1:**
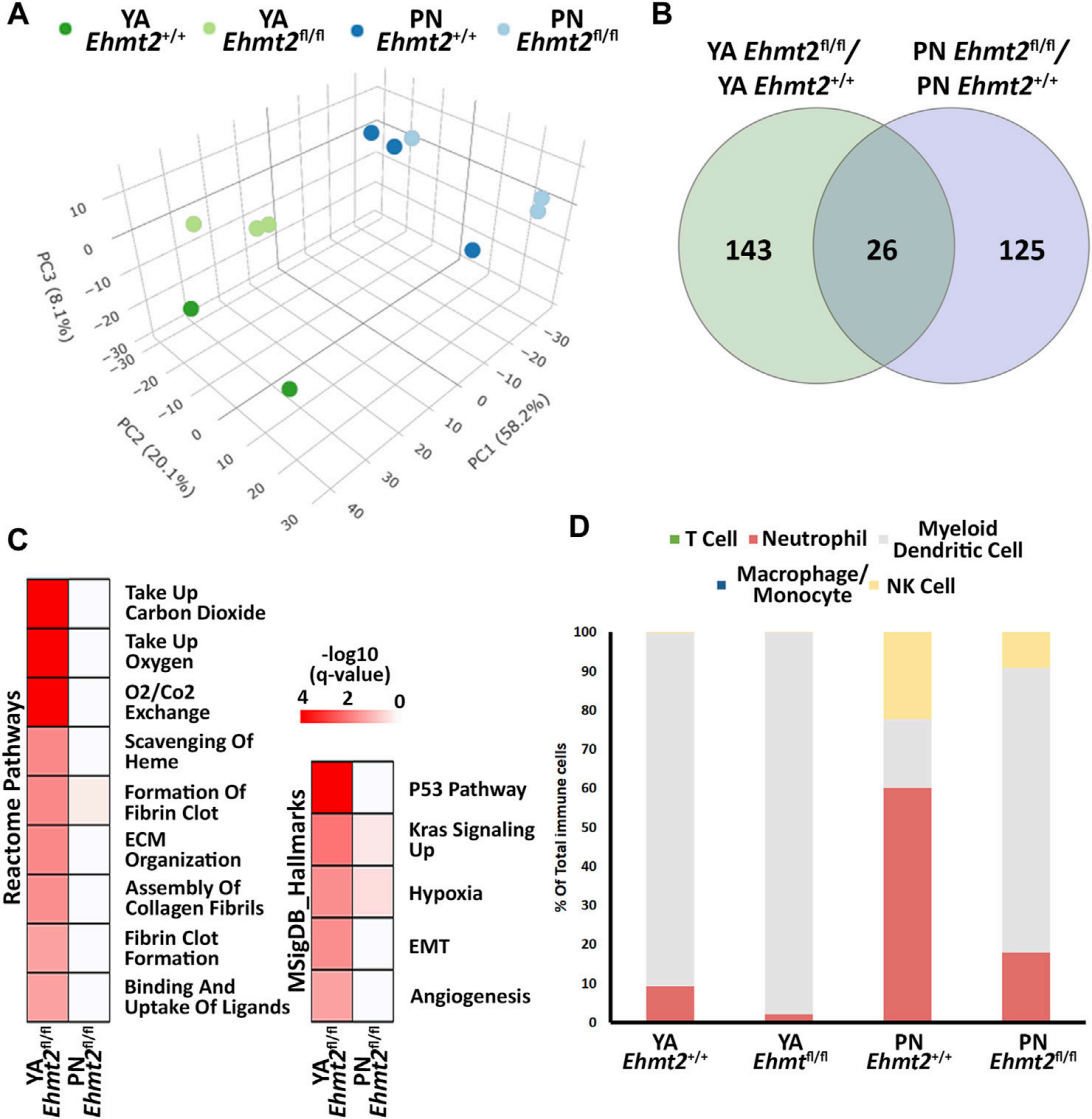
*Ehmt2* inactivation modulates the epigenetic landscape of mouse pancreas towards stress-related pathways. **(A)** Principal Component Analysis (PCA) based on Differentially Expressed Genes (DEGs) from RNA-seq conducted on pancreas tissue from mice at Postnatal (PN) day 10 and young adult (YA) 4–5 weeks, with and without Ehmt2, is shown. **(B)** Venn diagram illustrates the variation in DEGs upon *Ehmt2* knockout (*Ehmt2* ^*fl/fl*^) in the pancreas compared to *Ehmt2* wild-type (*Ehmt* ^*+/+*^) mice for YA and PN cohorts. **(C)** MSigDB Hallmarks and Reactome pathway enrichment analyses reveal the activation of functional pathways for DEGs in *Ehmt2* ^*fl*/fl^ animals compared to their respective *Ehmt2* ^+/+^ counterparts. **(D)** QuanTIseq deconvolution analysis predicts immune cell composition based on the percentage of total immune cells.

Previously, we reported that mice with pancreas-specific *Ehmt2* knockout develop and grow normally, adopting the right size, shape, and histology (Urrutia et al., 2021). While this phenotype indicates that this protein is not essential for organ development, it does underscore a function for Ehmt2 in regulating gene expression networks during pancreatic maturation and pancreatitis. We harvested pancreas from *Pdx1-Cre; Ehmt2* ^*fl/fl*^ (*Ehmt2* ^*fl/fl*^) and *Pdx1-Cre;Ehmt2* ^*+/+*^ (*Ehmt2* ^*+/+*^) mice at both postnatal (PN) day 10 and young adulthood (YA) 4–5 weeks of age and performed RNA-seq. Principal component analysis (PCA) shows a defined clustering of PN and YA *Ehmt2* ^*fl/fl*^ animals apart from their control groups (Figure 1A), which is more pronounced when the age groups are analyzed separately (Supplementary Figures S1A, B). We identified 143 and 125 unique differentially expressed genes (DEGs) between *Ehmt2* ^*fl/fl*^ and *Ehmt2* ^*+/+*^ for YA and PN, respectively, with 26 overlapping between both age groups (Figure 1B; Supplementary Table S1). We performed network enrichments to infer the function of the transcriptional landscape of the pancreas from these animals (Figure 1C). Upregulated genes showed significant enrichment for fibrin clot formation, hypoxia, and KRAS signaling up in the PN *Ehmt2* ^*fl/fl*^ mice compared to those with *Ehmt2* intact. The YA group was also enriched for these pathways and others, including erythrocyte gas exchange, extracellular matrix formation, and the scavenger receptor pathway. In contrast, no significant pathway enrichment was found among the downregulated DEGs. Comparison of molecular signatures in the YA upregulated DEGs group using MSigDB revealed enrichment of growth inhibitory pathways like P53 combined with KRAS signaling up, a phenomenon known to result in replication stress, cell cycle arrest, and apoptosis. More specifically, we found upregulation of key kinases involved in the P21 pathway, such as *Cdkn1a*, *Chek2* and *Ccng1*, which we have previously shown causes cell-cycle arrest in the presence of activated KRAS in acinar cells (Urrutia et al., 2021). Moreover, these changes were accompanied by hypoxia, angiogenesis, and epithelial-mesenchymal transition (Figure 1C). To evaluate fractions of specific immune cell types with the limited number of DEGs, particularly as these mice were not immuno-stimulated and thus expected to have a relatively low number of resident immune cells, we conducted a constrained deconvolution analysis using quanTIseq (Finotello et al., 2019). Even though this method cannot determine differences in absolute abundance of immune populations, this analysis revealed alterations in the relative proportions of certain immune cell types induced by *Ehmt2* inactivation at different developmental times. While the PN *Ehmt2* ^*fl/fl*^ pancreas had a 55.3% increase in dendritic cell populations along with 42.1% and 13.2% decreases in neutrophil and NK cells, respectively, we found that the mature pancreas at the YA stage became less variable between *Ehmt2* ^*+/+*^ and *Ehmt2* ^*fl/fl*^, with a 7.5% increase in marker genes for dendritic cells, a 7.3% decrease in neutrophil markers, and a minimal 0.2% decrease in NK cells (Figure 1D). Thus, we found that loss of *Ehmt2* most notably results in de-repression or upregulation of genes, in particular those that relate to growth inhibitory programs and cellular stress responses.

Next, we performed integrative analysis and functional modeling by using the natural language processing-based identification of gene sets relationships from the ShinyGO suite combined with semantic-based algorithms using the DEGs from the across the different transcriptional landscapes as input (Ge et al., 2020). This approach yielded several functional gene groups associated with pancreatic maturation in the *Ehmt2* ^*fl/fl*^ pancreas. For instance, we found higher representation of genes related to multiple diverse cellular functions including adhesion, extracellular matrix organization (ECM), inflammation response, stress, apoptosis, digestive enzymes, metabolism, transport, epigenetics, and cell motility (Table 1). We also noted a differential activation of the hemoglobin locus control region (LCR) for *Hba-a1, Hba-a2, Hbb-bs*, and *Hbb-bt*, which serves as a pathognomonic indicator of alterations in nuclear 3D organization upon *Ehmt2* loss due the subsequent decrease of H3K9me2 domains associated with reorganization of the active compartment in the nucleus (Yan et al., 2020; Fukuda et al., 2021; Takase et al., 2023). Furthermore, transcription factor motif analysis identified key upstream regulators of all DEGs related to *Ehmt2* inactivation, among which we find main transcriptional regulators of several pathways congruent with the mSigDB functional enrichments (enrichment of transcription factor binding motifs in DEG promoters; Table 2). In summary, loss of Ehmt2 results in dysregulation of the epigenetic landscape affecting essential signaling pathways, such as p53 and KRAS, which are known to cause replication stress, arrest cell proliferation, and induce apoptosis, combined with changes in the immune cell populations, suggesting an important function for Ehmt2 in maintaining pancreatic homeostasis and a susceptibility of *Ehmt2* ^*fl/fl*^ mice to heightened injury-inflammation repair responses.

**TABLE 1:** Functional annotation of DEGs regulated by *Ehmt2* inactivation in young adult mice.

Functional groups	Number of genes	Gene list
Adhesion	13	Cdh13; Cldn2; Cldn6; Cldn9; Egflam; Fat3; Itgal; Itgb4; Nxph2; Thsd4; Tm4sf4; Tspan1; Vtn
Angiogenesis	1	Cd248
Apoptosis	5	Bex2; Pycard; Stk17b; Trib3; Wwox
Cell Cycle	4	G0s2; Ccng1; Cdkn1a; Chek2
Cluster	3	Obp2b; Olfml2a; Olfml3
Coagulation	3	F2; Fgl2; Tfpi
Cytoskeleton	7	Rsph9; Arhgap45; Arhgdib; Coro1a; Eppk1; Kif21b; Rhobtb1
Development	1	Evc
DNA Repair	1	Dnajc22
ECM	13	Adamts12; Cilp; Col18a1; Col1a1; Col5a2; Cp; Eln; Gdf10; Lamc3; Mfap4; Ptn; Sdc1; Sftpd
Endocrine	1	Chgb
ER Chaperone	1	Agr2
Growth Factor	2	Gpr39; Ntrk2
Immune	15	Azgp1; Chac1; Fcgbp; Lgals9; Ptprc; Scara3; Siglec1; Srgn; Trim35; Dock2; Dock8; Malt1; Peli1; Spp1; Cd74
LCR	4	Hba-a1; Hba-a2; Hbb-bs; Hbb-bt
Membrane	7	Emp2; Smim24; Tenm4; Tmem123; Tmem229a; Tmem45a; Tmprss4
Metabolism	35	Acp5; Alas2; Aldh1b1; Aldoc; Car2; Car8; Cdo1; Cyp4f14; Cyp4v3; Dglucy; Enpp2; Ephx1; Fabp5; Fnip2; Gc; Heph; Lcat; Ldhb; Lox; Mgam; Mgmt; Mgst1; Mia; Moxd1; Mttp; Ncald; Pamr1; Pdzk1ip1; Phlda3; Pigr; Pkig; Proc; Sod3; Sulf2; Ube2l6
Mitosis Chromatin	1	Rsrp1
Pseudogene	1	Gm4744
Regeneration	1	Reg3b
RNA	1	Plet1
Signaling	5	Syk; Bex4; Plcb4; Pld1; Ror1
Stress	6	Ambp; Cryab; Ddit3; Ddit4l; Osgin1; Sesn2
Tnf Family	3	C1qtnf6; Tnfrsf19; Traf3ip3
Transcription	9	Eda2r; Et v5; Jun; Kat8; Klf10; Zfc3h1; Zfp651; Zmat3; Zfp703
Transporter	11	Aqp1; Atp1b1; Kcnk5; Scn1b; Slc16a5; Slc1a2; Slc28a3; Slc39a4; Slc4a1; Slc5a1; Slc9a9
Vesicular Transport	1	Scamp5
Zymogen	13	1810009J06Rik; 2200002J24Rik; Amy1; Amy2a1; Amy2a3; Gm10334; Gm2663; Gm5771; Klhl13; Prss1; Prss3; Serpine2; Serpinf1

**TABLE 2:** Transcriptional factor enrichment of DEGs upon *Ehmt2* inactivation in young adult mice.

Enriched motif in promoter	TF	TF family	P val	FDR
GGGGGTGG	Zfp281	C2H2 ZF	7.0E-06	5.8E-03
GGGGGG	Zfp740	C2H2 ZF	4.1E-05	1.1E-02
GGGGGGGGGCC	Patz1	C2H2 ZF	1.5E-04	2.8E-02
GGGGCCCAAGGGGG	Plag1	C2H2 ZF	1.7E-04	2.8E-02
GGGG	Zfp202	C2H2 ZF	1.5E-03	1.4E-01
TGGGAATACC	Ikzf1	C2H2 ZF	1.8E-03	1.4E-01
AGGG	Sp110	SAND	1.8E-03	1.4E-01
CCACCTG	Atoh8	bHLH	1.9E-03	1.4E-01
AGGTGTGA	Mga	bHLH,T-box	2.1E-03	1.5E-01
CAGGTG	Tcf3	bHLH	2.9E-03	1.8E-01
AGGTGTGA	Tbx5	T-box	3.2E-03	1.8E-01
GTACC	Gm98	Ndt80/PhoG	3.5E-03	1.8E-01
TGACCTTGACTGACCT	Esrra	Nuclear receptor	3.8E-03	1.8E-01
GGG​GGG​GGG​TGG​TTT​GGG​G	Rreb1	C2H2 ZF	4.0E-03	1.8E-01
CAGGTG	Tcf12	bHLH	4.2E-03	1.8E-01
AGGTGTGA	Tbx4	T-box	5.2E-03	2.2E-01

### 3.2 *Ehmt2* inactivation in acinar pancreatic cells enhances the response to organ inflammation

**FIGURE 2:**
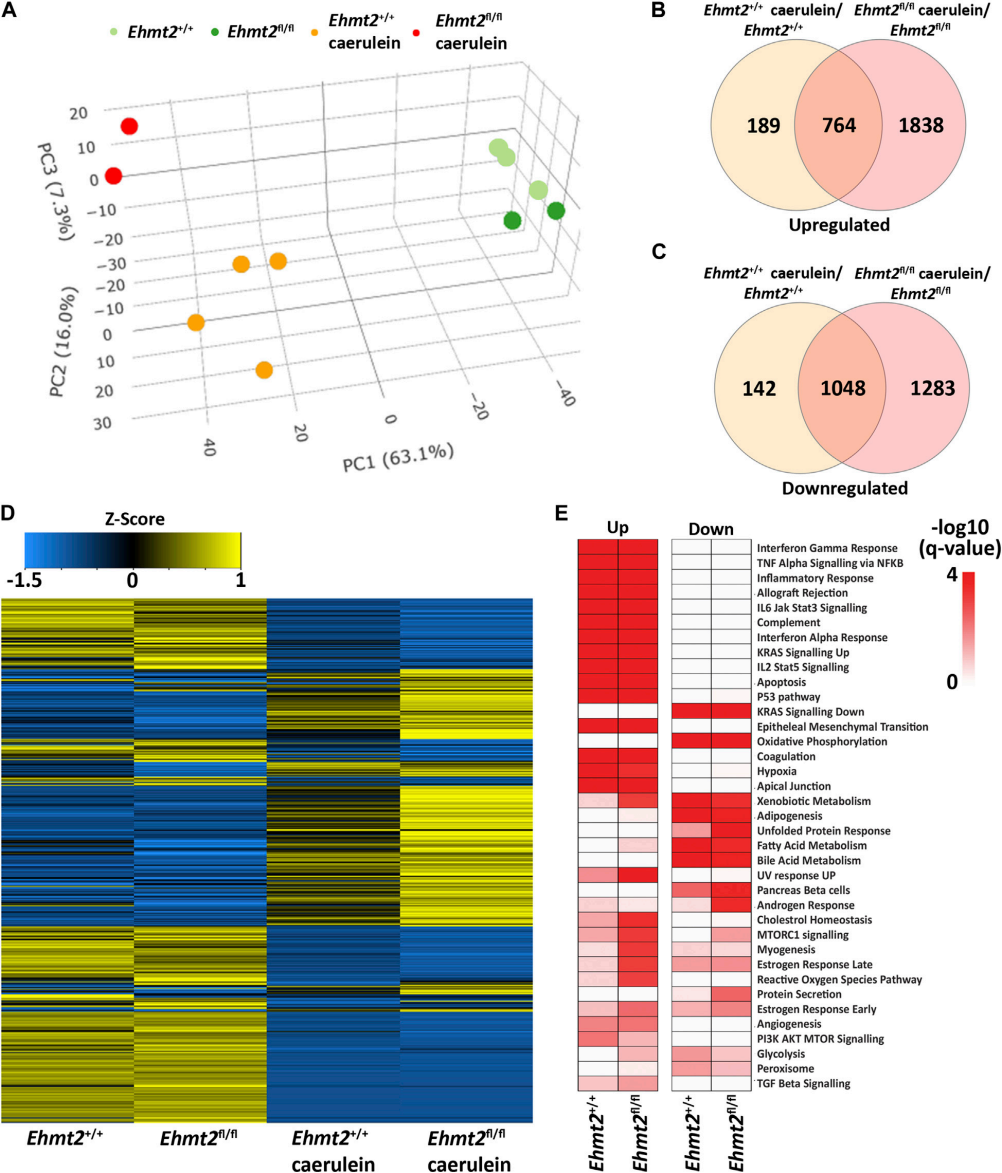
Ehmt2 is crucial for maintaining faithful transcriptional homeostasis in response to injury during acute pancreatitis. **(A)** PCA based on DEGs from RNA-seq conducted on pancreas tissue from *Ehmt2* ^*+/+*^ and *Ehmt2* ^*fl/fl*^ adult mice both with and without induction of acute pancreatitis is shown. Venn diagram illustrates the overlap in significant DEGs between mice treated with caerulein and untreated mice, comparing *Ehmt2* ^*+/+*^ and *Ehmt2* ^*fl/fl*^ animals for both **(B)** upregulated and **(C)** downregulated genes. **(D)** Heatmap displays the average expression of DEGs that were significant in at least one condition for *Ehmt2* ^*+/+*^ and *Ehmt2* ^*fl/fl*^ animals in absence or presence of caerulein treatment. **(E)** MSigDB Hallmarks pathway enrichment analysis of significant DEGs between mice treated with caerulein and untreated mice in both *Ehmt2* ^*+/+*^ and *Ehmt2* ^*fl/fl*^ animals is shown for upregulated and downregulated genes.

To evaluate the relative contribution of Ehmt2 to the epithelial cell response during pancreatic inflammation, we repeatedly injected *Ehmt2* ^*+/+*^ and *Ehmt2* ^*fl/fl*^ mice with caerulein to induce acute pancreatitis and performed RNA-seq. PCA analysis showed a clear separation of mice subjected to caerulein compared to their untreated counterparts (Figure 2A). Notably, the untreated samples, regardless of *Ehmt2* inactivation status, clustered closely together on one end of the PCA plot. In contrast, samples undergoing acute pancreatitis displayed a distinct separation between the *Ehmt2* ^*+/+*^ and *Ehmt2^fl/fl^* pancreatic samples (Figure 2A). Using pairwise analysis between caerulein and untreated groups, we found 5,263 DEGs. Of these DEGs, 1,812 were shared between *Ehmt2* ^*+/+*^ and *Ehmt2^fl/fl^* with acute pancreatitis, consisting of 764 upregulated and 1,048 downregulated DEGs (Figures 2B, C; Supplementary Table S2). In terms of unique DEGs, 1,838 were distinctly upregulated upon acute inflammation in the *Ehmt2^fl/fl^* mice versus 189 in the *Ehmt2* ^*+/+*^ group (Figure 2B). Correspondingly, 1,283 were exclusively downregulated during acute pancreatitis in the *Ehmt2^fl/fl^* animals compared to 142 in the *Ehmt2* ^*+/+*^ (Figure 2C). We used RT-qPCR to validate a set of upregulated and downregulated gene targets among the most significant DEGs identified in *Ehmt2* ^*fl/fl*^ animals with acute pancreatitis (Supplementary Figure S2). Interestingly, a large subset of the genes that were found in the *Ehmt2* ^*+/+*^ animals, which would constitute the response to acute pancreatitis, overlapped with the *Ehmt2^fl/fl^* mice (80% of upregulated and 91% of downregulated genes). Moreover, 70% of upregulated genes and 55% of downregulated genes that were differentially regulated in *Ehmt2^fl/fl^* animals were unique to the loss of *Ehmt2*. This highlights that Ehmt2 plays a major role during acute pancreatitis in maintaining transcriptional homeostasis in response to injury. Through heatmap clustering analyses of these DEGs, we identified that the genes differentially regulated in both *Ehmt2* ^*+/+*^ and *Ehmt2^fl/fl^* animals presented with an amplified response when *Ehmt2* was inactivated (Figure 2D). This finding in the *Ehmt2^fl/fl^* mice with acute pancreatitis is congruent with a principal role of Ehmt2 in mediating gene expression in response to tissue injury. In our analysis of the upregulated and downregulated genes, we found that *Ehmt2* ^*+/+*^ and *Ehmt2^fl/fl^* mice with acute pancreatitis exhibited significant enrichment of similar pathways, specifically genes involved in injury-inflammation through activation of the oncogenic KRAS, P53, TNFα and TGF-β signaling pathways (Figure 2E). To delve deeper, we manually examined genes upregulated in acute pancreatitis compared to untreated mice, focusing on the subset that were uniquely activated or further de-repressed during the inflammatory response upon *Ehmt2* inactivation. This analysis revealed a network of upstream regulatory genes that include *Il1b*, *Il1r1*, *Tnf*, *Csf3r* and members of the Ccl family (summarized in Table 3), which are known recruit and activate immune cells. With Il1r1 serving as the primary receptor for Il1b, this pathway is recognized for its role in triggering NF-κB signaling pathway activation, which we also found. We also detected increased gene expression networks related to immune cell activation and function, such as *Slamf* and cell surface molecules receptors. Similarly, the upregulation of chemokine signaling by C-X-C motif chemokine ligand families, indicates a heightened recruitment and migration of immune cells to inflamed pancreatic tissue. Notably, we also found upregulation of integrin and Interleukin cytokines, with roles in immunomodulatory processes that influence the balance between pro-inflammatory and anti-inflammatory responses. Thus, the increased expression of various inflammatory mediators highlights a cascade of cellular events that seem to culminate in the initiation and propagation of the enhanced immune response discovered in the *Ehmt2* ^*fl/fl*^ mice. In addition, there was increased representation of genes involved in cytoskeleton and cell adhesion, transcriptional regulation, DNA replication and repair, metabolic and oxidative stress, and RNA processing that collectively function to enhance cell survival, proliferation, and tissue regeneration during pancreatitis (Table 3). Contrastingly, the downregulated genes showed substantial enrichment of metabolic and proteostasis pathways, such as fatty and bile acid metabolism and unfolded protein response. This observation highlights that acute pancreatitis causes drastic changes to the pancreas gene expression landscape, reflecting that normal functionality of the pancreas is reduced (Figure 2E). Furthermore, we observed an increased enrichment of genes involved in a repair response, such as activation of mTORC1, PI3K-Akt, androgen and estrogen hormone signaling pathways. Lastly, we observed activation of genes involved in other signaling networks to help the pancreas tissue cope with damage caused by acute pancreatitis, such as angiogenesis to compensate for increased metabolic demand (Figure 2E). Notably, in the *Ehmt2* ^*fl/fl*^ pancreas tissues, the enrichment and overall number of genes for each ontology was found to be of greater significance with an increased number of genes in each of the categories, specifically for the UV response, cholesterol, mTORC1, myogenesis, estrogen, and reactive oxygen related pathways (Figure 2E). Thus, this data further highlights the Ehmt2-mediated functions that are necessary for the injury-inflammation-repair response during acute pancreatitis.

**TABLE 3:** Enhanced expression/de-repression of Ehmt2-regulated genes that are associated with the inflammatory and immune response in acute pancreatitis.

Functional groups	Number of genes	Gene list
Inflammation and Immune Response	56	***Il1b***; Il6; Il18; Tnf; Ccl2; Ccl5; Cxcl1; Cxcl2; Cxcl10; Ccl20; Ifng; Saa3; Nos2; Ptgs2; Hmox1; Lcn2; Cd14; C3; Ly6g; Cd68; Cd86; Fcgr3; Msr1; Fcer1g; Ccr2; Il10ra; Il10rb; ***Il1r1***; Il1rap; Il1rl1; Il27ra; Ifngr1; Ifngr2; Tnfrsf1b; Csf1r; Csf2rb; Csf2ra; Gss; Paqr7; Cstb; Nnmt; Anxa11os; Gm49776; Dusp18; Rbm12; Atp11a; Myo1c; Gja1; Pxn; Synpo; Epo; Capza2; Mybl2; Dbn1; Srgap1; Snx2
Cytoskeleton and Cell Adhesion	44	Actb; Actg1; Acta2; Vim; Cdh1; Cdh2; Cdh5; Itga1; Itga2; Itga3; Itga5; Itgb1; Itgb3; Itgb4; Itgb5; Vcl; Parva; Tln1; Tln2; Actn1; Actn2; Actn3; Actn4; Cfl1; Cfl2; Capg; Capn1; Capza1; Capza2; Cldn2; Uhrf1bp1l; Klhl5; Cpsf4; Pdlim5; Sept7; Mpp7; Pxn; Sept10; Ctnnd1; Col4a1; Col4a2; Col5a1; Col5a2; Col6a1
Transcription Factors and Regulators	40	Jun; Fos; Stat3; Stat1; Crebbp; Ep300; Cebpb; Cebpd; Cebpe; Cebpg; Cebpa; Junb; Jund; Fosb; Fosl1; Fosl2; Stat2; Stat4; Stat5a; Stat5b; Stat6; ***Nfkbia***; ***Nfkb1***; ***Nfkb2***; ***Rela***; ***Relb***; Mef2a; Gtf2f1; Elk4; Bcap29; Elk3; Xrn2; Nfatc4; Heg1; Zfp90; Klf13; Tnfrsf1a; Gm17046; Axl; Gm49711
Cell Cycle and DNA Replication/Repair	15	Ccna1; Ccnb1; Ccnb2; Ccne1; Ccne2; Cdk1; Cdk2; Cdk4; Cdk6; Cdkn1a; Cdkn1b; Cdkn2a; Cdkn2b; Rb1; E2f1
Metabolism and oxidative stress	28	Sod1; Sod2; Cat; Gpx1; Gpx2; Gpx4; Gpx7; Gpx8; Prdx1; Prdx2; Prdx3; Prdx4; Prdx5; Prdx6; Nqo1; Gss; Gsta1; Gsta2; Gstm1; Gstm2; Gstm3; Gstm4; Gstm6; Gstm7; Gsto1; Gsto2; Gstp1; Gstt1
RNA Processing and Splicing	30	Ddx5; Ddx17; Dhx9; Prpf3; Prpf4; Prpf6; Prpf8; Ddx39b; Ddx41; Ddx54; Dhx15; Dhx40; Dhx57; Dhx58; Dhx8; Ddx21; Ddx46; Ddx47; Ddx50; Ddx52; Ddx53; Ddx55; Ddx58; Dhx30; Dhx32; Dhx33; Dhx34; Dhx35; Dhx37; Dhx38

**FIGURE 3:**
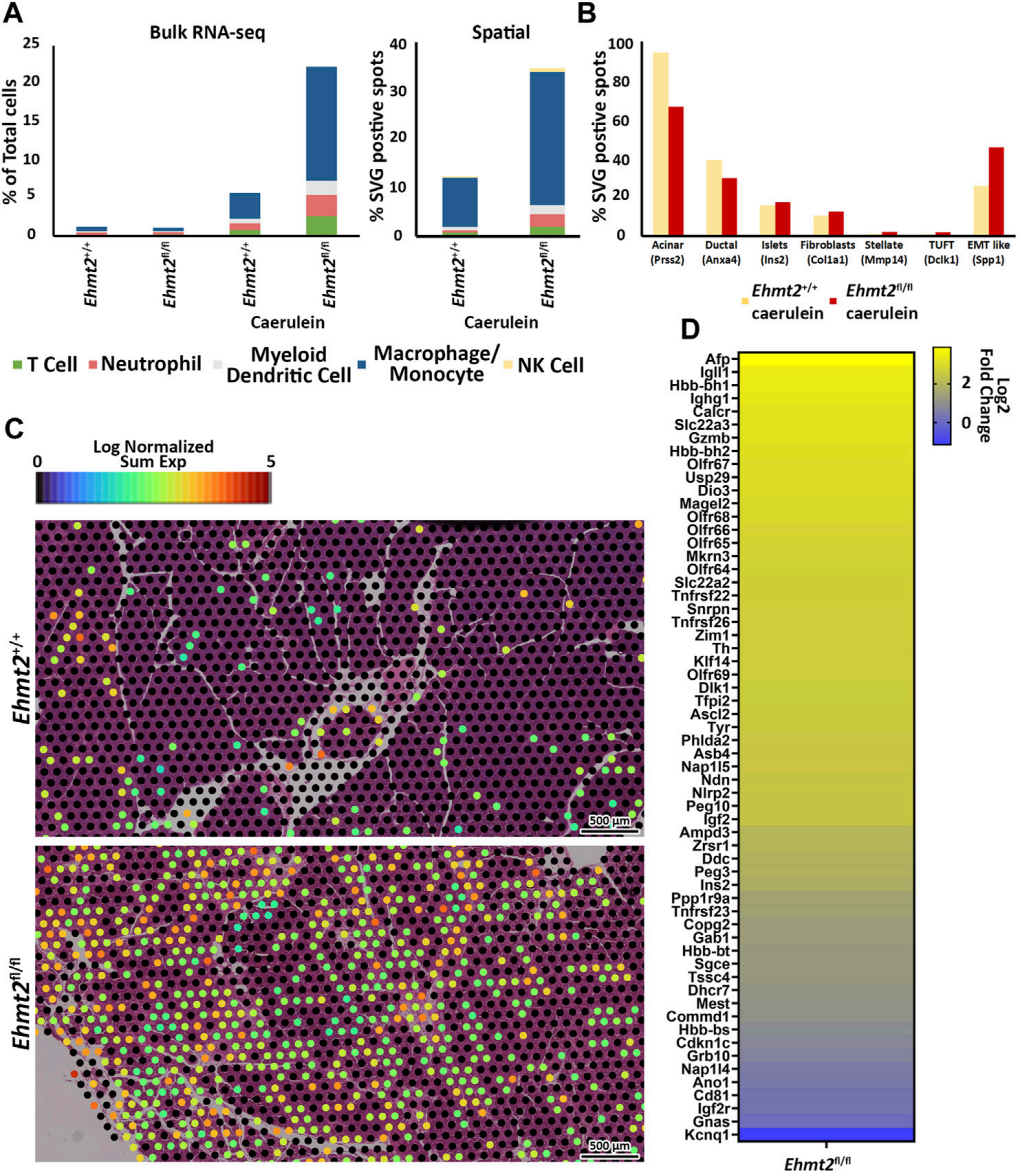
*Ehmt2* deficiency promotes immune cell infiltration in injured pancreatic tissue. **(A)** MCPcounter deconvolution analysis predicts immune cell composition in the total cell population using Bulk RNA-seq data from *Ehmt2* ^*+/+*^ and *Ehmt2* ^*fl/fl*^ animals, in the absence or presence of caerulein treatment. Manual spatial deconvolution is conducted using spots positive for SVGs and stringent immune cell markers in pancreas samples from caerulein-treated *Ehmt2* ^*+/+*^ and *Ehmt2* ^*fl/fl*^ animals. **(B)** Bar graph shows pancreas cell deconvolution utilizing spots positive for SVGs of markers as indicated for a subset of pancreatic cells in pancreas tissue from caerulein-treated *Ehmt2* ^*+/+*^ and *Ehmt2* ^*fl/fl*^ animals. **(C)** Visualization of macrophage SVG-positive spots is overlayed on caerulein-treated *Ehmt2* ^*+/+*^ and *Ehmt2* ^*fl/fl*^ pancreas samples. **(D)** Heatmap displays log2 fold change for 60 genes found regulated by locus control regions and imprinted control regions.

To reveal differences in the composition of the immune cell infiltrate across the total cells found between the *Ehmt2* ^*+/+*^ and *Ehmt2^fl/fl^* pancreas of caerulein-treated animals, we used MCP Counter, an expanded deconvolution method due to the increased inflammation under these conditions (Figure 3A). The induction of acute pancreatitis highlighted immune cell signaling patterns that distinguished *Ehmt2* ^*+/+*^ from *Ehmt2^fl/fl^*, also revealing an enhanced immune response when Ehmt2 function is lost with the number of markers for immune cells increasing by a total of 16.6%. Specifically, we identified an increased signal of 1.9% for T-cells in animals with *Ehmt2* inactivation. Similar increases were found for canonical markers of neutrophils and myeloid dendritic cells with an increase of 1.9% and 1.2%, respectfully, for *Ehmt2^fl/fl^* animals compared to *Ehmt2* ^*+/+*^ mice. Notably, macrophages represented the most substantial increase of 11.6% upon *Ehmt2* knockout. (Figure 3A). These findings highlight the important role of Ehmt2 in regulating immune cell infiltration during acute pancreatitis. Thus, we used spatial transcriptomics to validate our findings from bulk RNA-seq. Spatial transcriptomics technologies, while unable to achieve single-cell resolution due to capturing gene expression data from regions containing on average 5-10 cells each, offer valuable insights by maintaining the spatial context within tissue samples (Grisanti Canozo et al., 2022). Analytically, we applied Seurat’s functionalities to define spatially variable genes (SVG) across tissue samples. To deconvolute the cell types within the spatial transcriptomic spots, we employed marker genes obtained from Zhou et al. (2022) and CellMarker (Zhang et al., 2019). We calculated the average number of spots containing SVGs indicative of immune infiltration by T cells (*Cd3e*), neutrophils (*S100a8*), dendritic cells (*Ccr7*), macrophages (*Apoe*), and NK cells (*Nk7g*) for *Ehmt2* ^*+/+*^ and *Ehmt2^fl/fl^* mice. In concordance with our bulk RNA-seq data, we detected the enhanced immune response in *Ehmt2^fl/fl^* compared to the *Ehmt2* ^*+/+*^ mice with comparable distributions of specific immune subtypes (Figure 3A). An average of 7,865 spots for *Ehmt2* ^*+/+*^ and 7,542 for *Ehmt2^fl/fl^* mice were assigned in total across the tissue, and from these total spots, 0.8% contained SVG expression indicative of T-cell infiltrate in the *Ehmt2* ^*+/+*^ pancreas, which increased to 1.9% in *Ehmt2^fl/fl^*. Similarly, we found higher proportions of markers for neutrophils and dendritic cells in *Ehmt2^fl/fl^* tissue (2.6% and 2.0%, respectively) compared to *Ehmt2* ^*+/+*^ (0.5% and 0.6%, respectively). Once again, the macrophage population signified the most striking change between *Ehmt2* ^*+/+*^ tissue (10.0%) and *Ehmt2^fl/fl^* (27.4%). Conversely, the difference in the proportions of NK cells were modest with 0.3% in *Ehmt2* ^*+/+*^ to 0.8% in *Ehmt2^fl/fl^* (Figure 3A). To more comprehensively address the complex pathology of pancreatic disease, we expanded our spatial transcriptomic analysis to other relevant cell types using previously reported markers as labeled in Figure 3B (Olaniru et al., 2023). We found a significant reduction in spots associated with the SVG marker for acinar cells (*Prss2*) in *Ehmt2^fl/fl^* samples, with only 66.6% of spots indicative of harboring acinar cells (Figure 3B). In contrast, *Ehmt2* ^*+/+*^ tissues displayed a more typical distribution, with 94.4% of spots representing the presence of acinar cells. Similarly, *Ehmt2^fl/fl^* exhibited a decrease in duct cells compared to *Ehmt2* ^*+/+*^ (39.1% vs. 30.0%). On the other hand, features for islets (*Ins2*) (15.9% vs. 17.4%), fibroblasts (*Cola1a*) (10.6% vs. 12.7%), stellate cells (*Mmp14*) (1.1% vs. 2.3%), and tuft cells (*Dclk1*) (1.0% vs. 2.2%) showed slight increases when comparing *Ehmt2^+/+^* tissues to *Ehmt2^fl/fl^*. Lastly, we evaluated SVGs that are hallmarks of epithelial-mesenchymal transition (EMT), given that this process is closely linked to pro-fibrotic signaling and activation of pancreatic stellate cells (Tian et al., 2016). Focusing on the gene *Spp1*, we found 25.7% of spots positive for EMT-like SVG expression in *Ehmt2* ^*+/+*^ mice with acute pancreatitis, which notably increased to 45.7% in *Ehmt2^fl/fl^* pancreas (Figure 3B). As the macrophage marker showed the highest increase across the immune cells, we correspondingly found a clear increase of high expressing *Apoe* spots, suggesting a higher infiltrate and immune response when *Ehmt2* is inactivated (Figure 3C). Thus, the results from spatial transcriptomics fundamentally confirmed our findings in bulk RNA-seq that *Ehmt2* loss in the acinar cell triggers an increased immune infiltrate upon induction of acute pancreatitis.

Subsequently, we compiled a well-categorized list of genes known to be regulated by chromatin structure and nuclear organization, including several LCR and Imprinted Control Region (ICR) genes and evaluated the log2 FC by spatial transcriptomic analysis, comparing *Ehmt2^fl/fl^* to *Ehmt2* ^*+/+*^ mice (Supplementary Table S3) (Moon and Ley, 1990; May and Enver, 1995; Terajima et al., 1995; Johnson et al., 1999; Yui et al., 2001; Li et al., 2002; Kajiyama et al., 2006; Yang and Corces, 2011; Thakur et al., 2016). This analysis revealed significant dysregulation of 60 LCR/ICR-controlled genes, specifically for LCRs such as *Afp*, *Gzmb*, and *Hbb*, and several imprinted genes in *Ehmt2^fl/fl^* mice. Notably, the well-studied Chr7 ICR1 and ICR2, known to control allelic expression of several genes in that region such as *Kcnq1, Cdkn1c, H19,* and *Igf2*, exhibited complete loss of preferential allelic expression, with both maternal and paternal genes showing derepression, except for *Cd81* and *Kcnq1*, suggesting disruption of chromatin structure and nuclear organization (Figure 3D). This looping mechanism plays a crucial role in controlling gene expression by altering active and inactive compartments of topologically associating domains in 3D. These collective findings suggest that Ehmt2 helps preserve the epithelial characteristics of acinar and ductal cells in response to pancreatic injury, particularly during instances such as acute pancreatitis. This involvement is underscored by its role in gene repression and the regulation of 3D nuclear localization, emphasizing the multifaceted mechanisms through which Ehmt2 contributes to the maintenance of cellular identity in the context of physiopathological stimuli.

### 3.3 Ehmt2-mediated pathways during pancreas inflammation are model-independent

**FIGURE 4:**
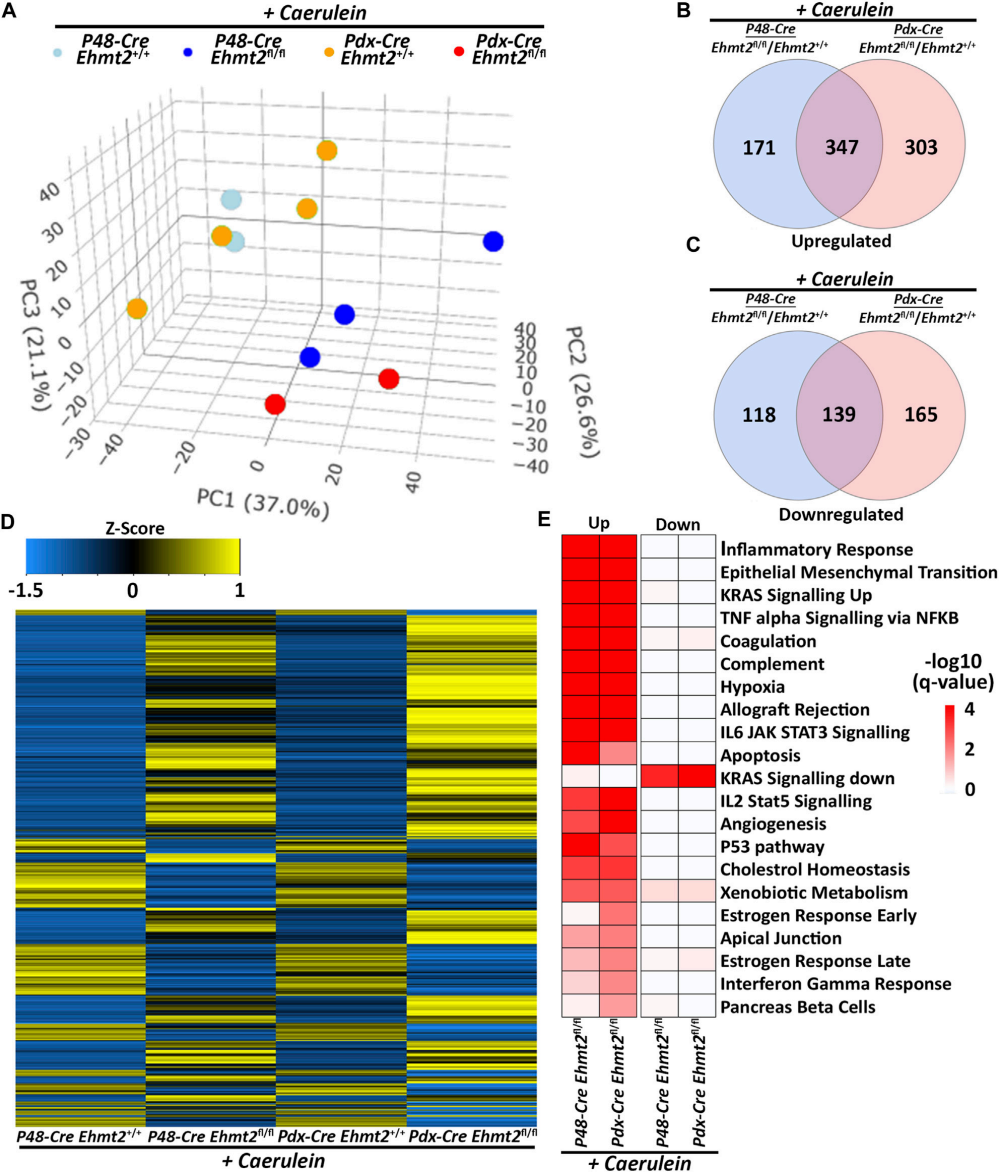
Ehmt2-mediated transcriptional regulation during acute pancreatitis is model independent. **(A)** PCA based on DEGs from RNA-seq conducted on pancreas tissue from adult *Ehmt2* ^*+/+*^ and *Ehmt2* ^*fl/fl*^ mice with acute pancreatitis for both *Pdx1-Cre* and *P48-Cre* driven models is shown. Venn diagram illustrates the overlap in significant DEGs between pancreatitis-induced *Pdx1-Cre* and *P48-Cre* models with *Ehmt2* inactivation compared to their *Ehmt2* ^*+/+*^ counterparts for **(B)** upregulated and **(C)** downregulated genes. **(D)** Heatmap displays the average expression of DEGs that were significant in at least one condition for *Ehmt2* ^*+/+*^ and *Ehmt2* ^*fl/fl*^ animals treated with caerulein in both *Pdx1-Cre* and *P48-Cre* models. **(E)** MSigDB Hallmarks pathway enrichment analysis is shown for significant DEGs between caerulein-treated *Pdx1-Cre* and *P48-Cre* models in both *Ehmt2* ^*+/+*^ and *Ehmt2* ^*fl/fl*^ animals for both upregulated and downregulated genes.

To confirm the enhanced tissue damage response during acute pancreatitis observed with loss of *Ehmt2*, we performed a pairwise analysis of the pancreatitis transcriptional landscape of the *Ehmt2* knockout generated either with the *Pdx1-Cre* (*Pdx1-Ehmt2* ^*fl/fl*^) or *P48* ^*Cre/+*^ (*P48-Ehmt2* ^*fl/fl*^) -driven models. This analysis aimed to identify genes involved in the injury response during acute pancreatitis that are transcriptionally regulated by Ehmt2, independent of the model used. PCA demonstrated that animals with intact *Ehmt2* clustered together during the acute pancreatitis treatment regardless of the model, *Pdx1* or *P48* ^*Cre/+*^, and separated from their *Ehmt2* ^*fl/fl*^ counterparts (Figure 4A). Analysis of both models together revealed 775 DEGs with the *P48-Ehmt2* ^*fl/fl*^ and 954 DEGs with the *Pdx1-Ehmt2* ^*fl/fl*^ model compared to their *Ehmt2* ^*+/+*^ counterparts (Figures 4B, C). Across DEGs, 486 genes were shared among *Ehmt2* ^*fl/fl*^ mice with acute pancreatitis, regardless of the Cre-driver used (Figures 4B, C; Supplementary Table S4). Specifically, we found 650 upregulated DEGs in the *Pdx1-Ehmt2* ^*fl/fl*^ and 518 with the *P48-Ehmt2* ^*fl/fl*^, of which 347 DEGs were the same between the two models (Figure 4B). For downregulated DEGs, there were 139 DEGs in common between both models from the 304 downregulated DEGs in *Pdx1-Ehmt2* ^*fl/fl*^ and 257 in the *P48-Ehmt2* ^*fl/fl*^ tissues (Figure 4C). The reproducibility and distinct separation between acute pancreatitis-induced animals with *Ehmt2* intact and those with *Ehmt2* loss, regardless of the Cre model, present in the PCA (Figure 4A) are also evident in the heatmap (Figure 4D). Ontological analysis of DEGs revealed significant enrichment of distinct functional groups, including inflammatory response and immune signaling, cell signaling and proliferation, as well as metabolism and hormonal regulation. For instance, upregulated DEGs in both the *Pdx1-Ehmt2^fl/fl^* and *P48-Ehmt2^fl/fl^* acute pancreatitis groups, that were classified within the inflammatory response and immune signaling functional group, were enriched in pathways such as TNFα signaling via NF-kB, interferon gamma response, allograft rejection, IL-6/JAK/STAT3, and IL-2/STAT5 signaling. For the cell signaling and proliferation functional group, we found enrichment in KRAS signaling up, EMT, angiogenesis, the p53 pathway, and apoptosis, cholesterol homeostasis, xenobiotic metabolism, estrogen response late, and estrogen response early were enriched under metabolism and hormonal regulation DEGs for the *Pdx1-Ehmt2^fl/fl^* mice. Similar enrichment was observed for upregulated DEGs in *P48-Ehmt2^fl/fl^* mice. Conversely, downregulated DEGs in the *Pdx1-Ehmt2^fl/fl^* and *P48-Ehmt2^fl/fl^* acute pancreatitis models were only significantly enriched for networks that signal KRAS down (Figure 4E). Moreover, we performed MCP Counter deconvolution to evaluate the immune cell infiltrate of the *P48-Ehmt2^+/+^* and *P48-Ehmt2^fl/fl^* animals with acute pancreatitis (Supplementary Figure S3A), finding similar changes with *Ehmt2* knockout as the *Pdx1-Cre* acute pancreatitis model (Figure 3A), namely, an increase in markers for macrophages, T-cells, and neutrophils. To further investigate the role of Ehmt2 in regulating key transcriptomic responses in pancreatitis, we concentrated on the 486 DEGs consistent across both models. We considered these DEGs as core genes regulated by Ehmt2 and used ChIP-Atlas, a comprehensive data-mining suite for exploring epigenomic landscapes, to access publicly available datasets (Zou et al., 2022). From this resource, we identified 15,851 target genes associated with Ehmt2 in both human and mouse genomes. Notably, 266 of the 486 DEGs (55%) have been recognized as *bona fide* targets of Ehmt2 (Supplementary Table S5). Moreover, pathway enrichment analysis of this subset of 266 confirmed Ehmt2 targets closely mirrors the significant enrichment observed in the core genes regulated by Ehmt2, including pathways involved in cell signaling and proliferation, EMT, and inflammation (Supplementary Figure S3B). Thus, whether *Ehmt2* is deleted from the pancreas using either the *Pdx1-Cre* or *P48* ^*Cre/+*^ model, the modified transcriptional impact of acute pancreatitis compared to their counterparts with *Ehmt2* intact are similar, highlighting that Ehmt2 participates primarily in preventing an enhanced injury-inflammatory repair response in this organ.

Lastly, we performed further analysis using NLP and semantic-based algorithms on DEGs identified by the *Pdx1* and *P48* models to determine shared pathways, which included signal transduction and immune defense (Table 4). We found enrichment in cell adhesion and extracellular matrix (ECM) genes, indicating changes in ECM organization, tissue remodeling processes, and immune cell trafficking within the pancreas during pancreatitis. Moreover, *Ehmt2* ^*fl/fl*^ animals upregulated several enzymes, transporters, and channels, which likely reflects the increased metabolic needs (Table 4). Additionally, we noted that derepression of *Il1b* and its known receptor *Il1r1*, as well as increased expression of upstream regulators involved in the NF-κB immune-mediated response were present in both the *Pdx1* and *P48* models. Analysis of enriched cis-regulatory elements within promoter regions of these DEGs revealed associations with universal stripe factors such as ZFP281, PATZ1, SP, and KLF5 among others (Zhao et al., 2022). This suggests that Ehmt2 may play a role in the crosstalk mechanism between these universal stripe factors, facilitating chromatin accessibility (Table 5). Thus, the response to inflammation that we describe here for loss of Ehmt2 in the pancreas epithelial cell is primarily not mediated by tissue enriched Stripe Factors but rather general Stripe factors that drive responses in many organs, suggesting that these processes may have broader relevance.

**TABLE 4:** Integrative analysis by biological modeling of model-independent DEGs to determine shared Ehmt2-dependent pathways.

Functional groups	Number of genes	Gene list
Signal transduction networks	91	Abi3bp; Ahr; Apobr; Arrb2; C1qb; ***Cd14***; Cd300c2; Cdc42bpb; ***Cxcl2***; Dusp10; Dusp26; Dusp6; Fcer1g; Fcgr1; Fcgr2b; Fcgr3; Fcgr4; Fmo1; Fmo4; Fpr2; ***Gadd45a***; Gbp2b; Grk5; Htr5a; Igsf9b; Il17a; Il17f; ***Il1b***; ***Il1r1***; Il1r2; Il1rap; Il1rn; Il33; Il6; Msn; Notch2; P2ry14; P2ry6; Rab7b; Rhoa; Rhoj; Rhou; Siglece; Slc7a14; Spp1; ***Tlr4***; Tnf; ***Tnfrsf11a***; Tnfrsf1b; Tnfrsf21; ***Tnfsf14***; Vav1; Vav3; Vsir; Wnt7b; Xirp2; Zeb2; Actg1; Camk2b; Ccnd1; Cd44; Cdkn1a; Ctsl; Ezr; Fas; Flna; Fn1; Fzd1; Fzd3; Hbegf; Hif1a; Itga2; Itga5; Itgav; Itgb5; Lum; Mapk13; Mmp2; Mmp9; Msn; Pdcd4; Pik3r3; Plaur; Tgfb2; Thbs1; Timp3; Tlr4; Vav1; Vav3; Wnt4; Wnt7b
Extracelullar matrix/Cell adhesion	41	Adam12; Adam23; Adam8; Adipoq; Agrn; Bgn; Cd44; Cdh17; Cdh6; Col18a1; Fbln2; Fn1; Lgals12; Lum; Mfap5; Msr1; Plaur; Sema6b; Thbs1; Timp1; Tnc; Cd97; Cd99; Cldn10; Cldn18; Cldn2; Cldn23; Cldn4; Cldn5; Cldn6; Cldn8; Cntrl; Ctnnb1; Gjb1; Gjb4; Kirrel; Kirrel3; Ncstn; Nr0b2; Syne4; Vsig2
Enzymes involved in metabolism	26	Amy2a4; Amy2b; Aldh1l1; Akr1cl; Apoc2; Aqp12; B4galt6; Car11; C2cd2; Cfd; Cthrc1; Cyp1b1; Cyp2b10; Cyp2d10; Cyp2j5; Ddhd2; Gck; Hsd17b13; Hmgcs1; Oas1g; Pgam2; Pnlip; Pnliprp1; Pnliprp2; Pygl; Ttc9
Transporters and channels	31	Abcb9; Apobec1; C2cd4a; C8a; Cftr; Clca3a2; Cyb5a; Cyth4; Fxyd3; Kcna3; Kcnd2; Kcnj12; Kcnj4; Kcnq1; Kcnq1ot1; Kcnv1; Kif21a; Klb; Pcsk5; Slc12a8; Slc15a3; Slc16a3; Slc25a29; Slc25a35; Slc26a1; Slc38a3; Slc41a1; Slc7a7; Slc7a14; Slco2a1; Sult4a1
Endogenous transcription factor pathway	29	Ahr; Bhlha15; Brsk2; Creb3l1; Creb3l2; Fos; Fosl1; Fosl2; Foxa3; Foxp2; Gfi1; Gtf2ird1; Hif1a; Jun; Kdm5d; Lbh; Myod1; Nrros; Pbx3; Ppargc1b; Rbpj; Tbxas1; Tc2n; Tead2; Tead4; Tgif1; Zfp36; Zfp36l2; Zfp706

**TABLE 5:** Transcriptional factor enrichment of DEGs by Ehmt2 during acute pancreatitis.

Enriched motif in promoter	TF	TF family	P val	FDR
GGGGTGGGGGAGGGG	Zfp281	C2H2 ZF	1.3E-07	8.0E-05
GGGGGTGG	Zfp281	C2H2 ZF	1.9E-07	8.0E-05
GGGGGGGGGCC	Patz1	C2H2 ZF	1.1E-06	3.0E-04
GGGGGG	Zfp740	C2H2 ZF	4.7E-06	9.9E-04
GTGGGGGGG	Zfp740	C2H2 ZF	1.5E-05	2.2E-03
GGGGGGG	Zfp740	C2H2 ZF	1.6E-05	2.2E-03
GGGCGGGGC	Klf5	C2H2 ZF	4.4E-05	4.6E-03
GGGGGCGGGGC	Sp2	C2H2 ZF	4.4E-05	4.6E-03
GGGGGCGG	Sp4	C2H2 ZF	1.4E-04	1.3E-02
CGTGGGCG	Egr1	C2H2 ZF	4.3E-04	3.6E-02
GGGCG	Klf8	C2H2 ZF	4.8E-04	3.7E-02
GGGTGGGGC	Klf4	C2H2 ZF	6.3E-04	4.4E-02
GGGGGCGG	Sp1	C2H2 ZF	8.1E-04	5.2E-02
GGGGGGT	Zic5	C2H2 ZF	9.1E-04	5.4E-02
GGGTTCGAGGGT	Zfp524	C2H2 ZF	1.2E-03	6.3E-02
ATGCGGG	Gcm1	GCM	1.4E-03	6.3E-02
GGGCGTG	Klf12	C2H2 ZF	1.5E-03	6.3E-02
GGGGGC	Zbtb7b	C2H2 ZF	1.5E-03	6.3E-02
CAGGTGAG	Zeb1	C2H2 ZF,Homeodomain	1.5E-03	6.3E-02
GGGCGTG	Klf7	C2H2 ZF	1.6E-03	6.3E-02
GGGGTGGTGG	Dnajc21	C2H2 ZF	1.6E-03	6.3E-02
GGGG	Zfp202	C2H2 ZF	1.7E-03	6.3E-02
GGG​GGG​GGG​TGG​TTT​GGG​G	Rreb1	C2H2 ZF	1.9E-03	7.1E-02
GGGGAT	Mzf1	C2H2 ZF	2.2E-03	7.6E-02
GGGTGGGGC	Klf4	C2H2 ZF	2.6E-03	8.5E-02
TGGGCA	Hic2	C2H2 ZF	2.6E-03	8.5E-02
GGGGGCGG	Sp4	C2H2 ZF	3.4E-03	1.0E-01
CGTGGGCG	Egr3	C2H2 ZF	3.4E-03	1.0E-01
GGGGAT	Mzf1	C2H2 ZF	3.8E-03	1.1E-01
TGGGTGTGGC	Klf1	C2H2 ZF	5.2E-03	1.4E-01

### 3.4 Morphological and biochemical assessment confirms the impact of *Ehmt2* inactivation on pancreas inflammation in both the *Pdx1*-and *P48*-driven models

**FIGURE 5:**
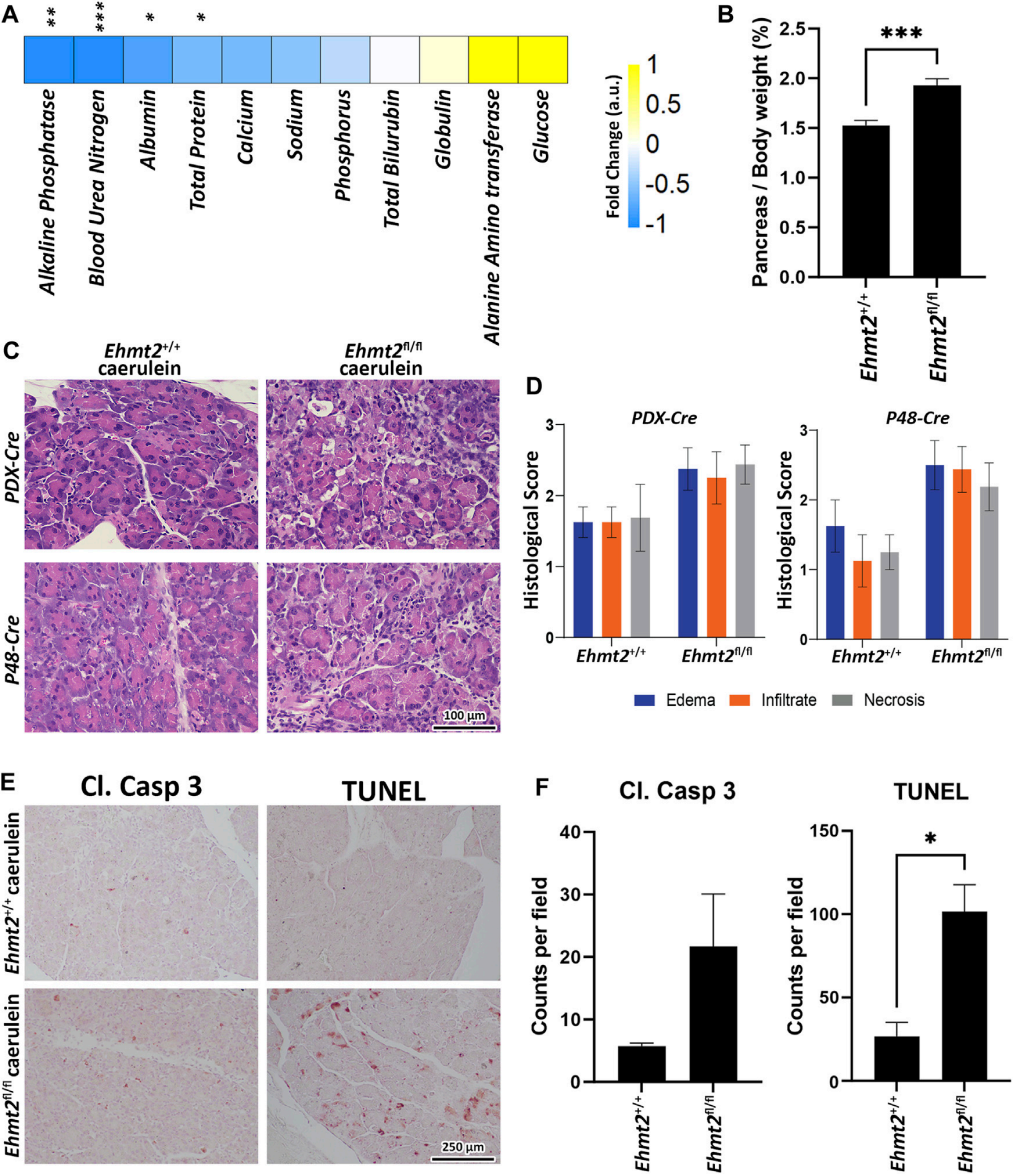
Ehmt2 deficiency in the pancreas epithelium increases tissue damage and deterioration in acute pancreatitis. **(A)** Heatmap shows the various circulatory markers in plasma of mice with acute pancreatitis. The difference in change between *Ehmt2* ^*+/+*^ and *Ehmt2* ^*fl/fl*^ groups have been depicted where +1 shows highest and −1 shows lowest difference in change. **(B)** Graph shows increased pancreas-to-body weight ratios after *Ehmt2* inactivation compared to control *Ehmt2* ^*+/+*^ animals in caerulein-induced acute pancreatitis. **(C)** Representative histology of caerulein-induced pancreatitis from *Pdx1-Cre* and *P48-Cre Ehmt2* ^*+/+*^ and *Ehmt2* ^*fl/fl*^ pancreas tissue is shown by hematoxylin-eosin-staining. Scale bar, 100 μm. **(D)** Pancreatitis histological scores for edema, infiltrate, and necrosis were assessed from four random fields (×20 objective) per slide, each containing at least 1,000 cells per field. Scores, expressed as mean ± SD scores, were calculated for each individual parameter. **(E)** Representative images are shown for immunohistochemical staining for cleaved Caspase three and TUNEL in pancreas tissue from *Pdx1-Cre* and *P48-Cre Ehmt2* ^*+/+*^ and *Ehmt2* ^*fl/fl*^ mice with caerulein-induced pancreatitis. **(F)** Bar graph depicts quantification for cleaved Caspase three and TUNEL assay reveals increased apoptosis in *Ehmt2* ^*fl/fl*^ compared to control *Ehmt2* ^*+/+*^ animals in caerulein induced pancreatitis. Plasma Samples; *n* = 6. Pancreas/Body weight samples; *n* = 3. Caspase/TUNEL assay; *n* = 6. (*, *p* < 0.05; **, *p* < 0.01; ***, *p* < 0.001; *t*-test).

Acute pancreatitis in humans is linked to both local and systemic complications. Systemic complications typically present as organ failure, observed in about 20% of cases (Garg and Singh, 2019). Thus, biochemical investigation was performed on serum from both *Pdx1-Cre* and *P48* ^*Cre/+*^ caerulein-treated mice with or without *Ehmt2* to evaluate liver and kidney function. We found significantly decreased levels of albumin, total protein, blood urea nitrogen (BUN) and alkaline phosphatase (ALP) levels in *Ehmt2* ^*fl/fl*^ animals compared to *Ehmt2* ^*+/+*^ mice (Figure 5A). The decrease in circulating albumin, coupled with heightened inflammation in *Ehmt2* ^*fl/fl*^ mice, suggests a potential increase in capillary permeability and the efflux of circulating albumin into interstitial spaces (Soeters et al., 2019), which is seen in cases of acute pancreatitis (Xu et al., 2023). The reduction in albumin, along with diminished total protein levels in *Ehmt2* ^*fl/fl*^ serum implies a potential impairment in liver and/or kidney function, frequently observed during acute pancreatitis episodes in humans (Li et al., 2017; Ocskay et al., 2021). Furthermore, decreased ALP levels in *Ehmt2* ^*fl/fl*^ serum may directly result from compromised liver function (Iluz-Freundlich et al., 2021). The alterations in serum constituents suggest that *Ehmt2* deficiency exacerbates inflammation under conditions of acute pancreatitis which can lead to multiple organ dysfunction and failure (Garg and Singh, 2019). Additionally, we found that the pancreas to body weight ratios increased when *Ehmt2* was inactivated during acute pancreatitis, likely resulting from a swollen and enlarged pancreas (Figure 5B). Indeed, histopathological examination of pancreatic tissue after induction of acute pancreatitis revealed that loss of *Ehmt2* from the pancreatic epithelium was characterized by an intensified reaction to caerulein administration with several notable histopathological changes compared to their *Ehmt2* ^*+/+*^ counterparts (Figure 5C). First, we detected a pronounced increase in inflammatory infiltrates, comprising a higher density of neutrophils, macrophages, and lymphocytes within the pancreatic parenchyma and interstitial spaces (Figure 5D). Additionally, we found more extensive edema with interlobular and intralobular spaces displaying marked expansion due to enhanced vascular permeability. Heightened vascular fragility and leakage were also evidenced by hemorrhagic areas in tissue from the *Ehmt2* ^*fl/fl*^ animals. Furthermore, acinar cells in *Ehmt2* ^*fl/fl*^ exhibited severe damage, with widespread cellular vacuolization, loss of cytoplasmic granularity, and cellular disintegration, leading to disrupted tissue architecture (Figure 5D). In contrast, acinar cells in *Ehmt2* ^*+/+*^ animals maintained a more intact structure even with the caerulein-induced inflammatory insult. The fibrosis was also notably heightened in *Ehmt2* ^*fl/fl*^ animals. Subsequently, we assessed whether this exacerbated response to acute pancreatitis in *Ehmt2* ^*fl/fl*^ animals was associated with acinar cell death. We found increased immunohistochemical staining for cleaved caspase three and TUNEL-positive cells, indicating increased apoptosis within the pancreatic tissue of *Ehmt2* ^*fl/fl*^ mice (Figures 5E, F). In summary, loss of *Ehmt2* in the pancreatic epithelium results in an augmented response to acute pancreatitis characterized by higher inflammatory cell infiltration, edema, acinar cell damage and death, as well as augmented necrosis.

**FIGURE 6:**
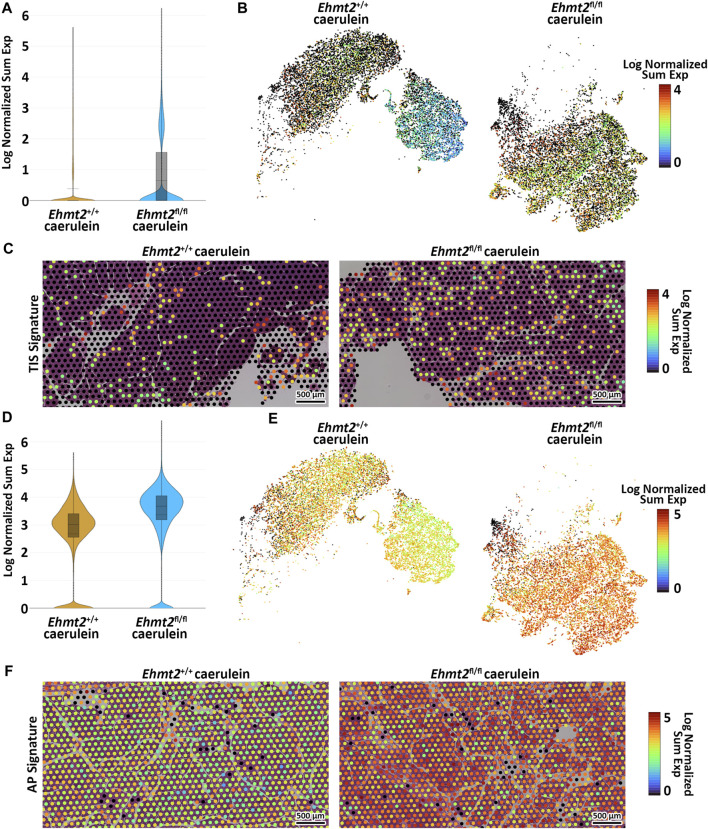
Loss of Ehmt2 results in derepression of tumor associated and acute pancreatitis inflammatory markers increasing susceptibility to tissue damage in the pancreas. **(A)** Violin plots display the log fold change of normalized gene expression, describing expression of the tumor inflammation signature (TIS) in spatial transcriptomics derived from pancreas tissues from acute pancreatitis induced *Ehmt2* ^*+/+*^ and *Ehmt2* ^*fl/fl*^ mice. **(B)** t-SNE plot illustrates the distribution of SVG positive spots corresponding to genes from the TIS. **(C)** Visualization overlays depict the spatial distribution and expression of TIS positive spots in acute pancreatitis samples from *Ehmt2* ^*+/+*^ and *Ehmt2* ^*fl/fl*^ mice. **(D)** Violin plots display the log fold change of normalized gene expression, describing expression of the acute pancreatitis (AP) signature in spatial transcriptomics derived from pancreas tissues of *Ehmt2* ^*+/+*^ and *Ehmt2* ^*fl/fl*^ mice with caerulein-induced acute pancreatitis. **(E)** t-SNE plot illustrates the distribution of SVG positive spots corresponding to genes from the AP signature. **(F)** Visualization overlays depict the spatial distribution and expression of AP signature positive spots in acute pancreatitis samples from *Ehmt2* ^*+/+*^ and *Ehmt2* ^*fl/fl*^ mice. Scale bars represent the log normalized sum expression of selected genes.

To validate the aggravated inflammatory response in *Ehmt2* ^*fl/fl*^ animals in comparison to the *Ehmt2* ^*+/+*^, we analyzed our spatial transcriptomics using a gene profile for the tumor inflammation signature (TIS) (Danaher et al., 2018; Loh et al., 2023). TIS expression in *Ehmt2* ^*+/+*^ was an average of 0.39, which increased to 0.65 in *Ehmt2* ^*fl/fl*^ pancreas tissues (Figure 6A). Overall, using a t-SNE plot, we found that 50.8% of SVG spots were TIS significant spots in the *Ehmt2* ^*fl/fl*^ tissues, and 45.3% were TIS significant in the *Ehmt2* ^*+/+*^ pancreas, of which most spots showed higher expression upon *Ehmt2* knockout (Figure 6B). We found that all genes from the TIS were statistically significant between the *Ehmt2* ^*fl/fl*^ pancreas compared to the *Ehmt2* ^*+/+*^ controls during acute pancreatitis, with a range from 0.34 for *Psmb10* as the least log2 FC to 3.48 for *Tigit* with the most significant change (Table 6). Moreover, the TIS-related SVGs were substantially increased in density when visualized overlaying the pancreas tissue for the *Ehmt2* ^*fl/fl*^ when compared to the *Ehmt2* ^*+/+*^ (Figure 6C). Using a gene signature for acute pancreatitis (Fang et al., 2022), we identified a marked increase of overall mean sum expression in *Ehmt2* ^*fl/fl*^ (3.3) animals in comparison to *Ehmt2* ^*+/+*^ (2.6) and an increase of 92% of positive SVGs for the *Ehmt2* ^*fl/fl*^ animals compared to 83% for the *Ehmt2* ^*+/+*^ mice (Figures 6D, E). Upon closer examination of individual genes, we observed that the genes in the signature for acute pancreatitis were predominantly overexpressed by a log2 FC of >0.8 in the *Ehmt2* ^*fl/fl*^ group compared to *Ehmt2* ^*+/+*^ (Table 6). Using the spatial visualization tool, we found substantial increased expression for each of the SVGs for the overall acute pancreatitis gene signature in the *Ehmt2* ^*fl/fl*^ pancreas compared to the *Ehmt2* ^+/+^ organ, concordant with *Ehmt2* inactivation enhancing the inflammatory response in acute pancreatitis (Figure 6F). Only three genes (*Hspb1*, *Krt8* and *Hsp90aa1*) exhibited higher expression in the *Ehmt2* ^*+/+*^ pancreas, albeit with a log2 FC no greater than 0.3. Thus, collectively, these results underscore the impact that *Ehmt2* inactivation within the pancreas epithelium has on the response to inflammatory injury of the whole organ.

**TABLE 6:** Spatial transcriptomics shows increased expression of TIS and acute pancreatitis signature markers in tissues from caerulein-treated *Ehmt2* ^*fl/fl*^ animals.

FeatureID	Signature	Feature name	G9a KO Log2 FC	*p*-value
ENSMUSG00000031897	Tumor Inflammation Signature	Psmb10	0.34	2.3E-02
ENSMUSG00000026104	Tumor Inflammation Signature	Stat1	0.67	1.0E-03
ENSMUSG00000035042	Tumor Inflammation Signature	Ccl5	0.80	3.3E-02
ENSMUSG00000035914	Tumor Inflammation Signature	Cd276	1.22	1.1E-14
ENSMUSG00000042190	Tumor Inflammation Signature	Cmklr1	2.64	5.9E-49
ENSMUSG00000016498	Tumor Inflammation Signature	Pdcd1lg2	2.79	2.4E-42
ENSMUSG00000004612	Tumor Inflammation Signature	Nkg7	2.89	3.4E-25
ENSMUSG00000016496	Tumor Inflammation Signature	Cd274	2.93	1.3E-47
ENSMUSG00000035000	Tumor Inflammation Signature	Dpp4	2.95	3.1E-35
ENSMUSG00000030124	Tumor Inflammation Signature	Lag3	3.02	4.6E-50
ENSMUSG00000048521	Tumor Inflammation Signature	Cxcr6	3.10	1.7E-41
ENSMUSG00000029417	Tumor Inflammation Signature	Cxcl9	3.12	3.8E-48
ENSMUSG00000031551	Tumor Inflammation Signature	Ido1	3.22	7.5E-27
ENSMUSG00000053977	Tumor Inflammation Signature	Cd8a	3.36	3.1E-43
ENSMUSG00000071552	Tumor Inflammation Signature	Tigit	3.48	3.1E-27
ENSMUSG00000004951	acute pancreatitis	Hspb1	−0.32	2.2E-02
ENSMUSG00000049382	acute pancreatitis	Krt8	−0.09	5.3E-01
ENSMUSG00000021270	acute pancreatitis	Hsp90aa1	−0.06	7.5E-01
ENSMUSG00000025283	acute pancreatitis	Sat1	0.81	2.7E-09
ENSMUSG00000115338	acute pancreatitis	Pnp	1.00	3.7E-14
ENSMUSG00000030342	acute pancreatitis	Cd9	1.08	5.3E-15
ENSMUSG00000007041	acute pancreatitis	Clic1	1.25	4.5E-22
ENSMUSG00000009687	acute pancreatitis	Fxyd5	1.56	3.0E-31
ENSMUSG00000022146	acute pancreatitis	Osmr	1.76	7.4E-26
ENSMUSG00000032231	acute pancreatitis	Anxa2	2.11	4.8E-62
ENSMUSG00000021091	acute pancreatitis	Serpina3n	2.31	6.0E-58
ENSMUSG00000026628	acute pancreatitis	Atf3	2.41	2.7E-62
ENSMUSG00000028494	acute pancreatitis	Plin2	2.83	1.7E-94
ENSMUSG00000005087	acute pancreatitis	Cd44	2.97	2.9E-125
ENSMUSG00000005413	acute pancreatitis	Hmox1	2.98	2.2E-105
ENSMUSG00000026822	acute pancreatitis	Lcn2	3.74	1.8E-171
ENSMUSG00000019122	acute pancreatitis	Ccl9	4.21	8.0E-243

## 4 Discussion

The current study offers significant insights into the role of Ehmt2 in pancreatic development and its response to inflammatory stimuli. Clinical and experimental observations have shown that individual responses to pancreatitis-causing stimuli vary, suggesting contributions from both genetic factors and environmental exposures (Weiss et al., 2021). However, the specific mechanisms underlying these individual responses, particularly concerning chromatin proteins that epigenetically regulate transcriptional landscapes, remain poorly understood. Thus, the current study was designed to directly investigate the role of Ehmt2, a robust transcriptional repressor, in experimentally induced acute pancreatitis. Utilizing a systems biology approach and genetically engineered mouse models, we employed RNA-Seq with deconvolution into a digital cytology approach, as well as spatial transcriptomics, to define the emergent properties resulting from the genetic inactivation of *Ehmt2* in pancreatic acinar cells. Our results reveal, for the first time, that *Ehmt2* inactivation increases the propensity of the normal pancreas to injury-inflammation, suggesting its crucial role in maintaining pancreatic homeostasis and moderating inflammatory responses. Notably, we observed significant transcriptional differences in *Ehmt2* ^*fl/fl*^ mice during postnatal and young adult stages, indicating the involvement of this epigenomic regulator in fine-tuning gene expression networks essential for pancreatic maturation. Furthermore, the induction of acute pancreatitis in *Ehmt2* ^*fl/fl*^ mice resulted in a more aggressive inflammatory reaction, highlighting its role in suppressing inflammatory gene networks and restraining the pancreatic inflammatory response. This enhanced response was consistently confirmed by morphological and biochemical evidence across different models, including both *Pdx1-Cre* and *P48* ^*Cre/+*^-driven models, indicating its robustness and independence from specific genetic backgrounds. However, a limitation of our analysis is that without a time-course study, it is unclear whether the enhanced inflammatory response with *Ehmt2* inactivation is due to alterations in the duration of the inflammatory response. Nevertheless, findings provide important insights into the molecular mechanisms underlying pancreatic diseases, particularly those with an inflammatory component, shedding light on the pathogenesis of these conditions.

The observation of enhanced inflammatory response in *Ehmt2* ^*fl/fl*^ mice holds significant implications from an evolutionary standpoint. The *Ehmt2* gene itself is embedded within a cluster of inflammatory genes (Scheer and Zaph, 2017), suggesting a potential evolutionary pressure that has preserved its role as a key regulator of inflammatory pathways across successive generations. This arrangement implies a functional importance of Ehmt2 in modulating inflammatory responses, with its genetic proximity to other inflammatory genes possibly indicating a coordinated regulatory mechanism. This evolutionary perspective underscores the critical role of Ehmt2 in maintaining homeostasis and regulating inflammatory processes within the pancreas and potentially other tissues.

Mechanistically, we identify key transcriptional nodes in epithelial cells that alter the profiles of secreted factors, impacting the reactions and function of surrounding cells. *Ehmt2* inactivation leads to an enhanced inflammatory response rather than a distinct one, evidenced by changes in the levels of chemokine family members crucial for leukocyte recruitment to inflammatory sites (Popiolek-Barczyk et al., 2020). This observation has broad implications for various diseases, including developmental disorders, inflammatory conditions, and cancer. Moreover, we found the increased expression of several inflammatory mediators, such as *Il1b*, *Il1r1*, *TNF* among others, that likely augment the activation of the NF-κB signaling pathway. Interestingly, Ehmt2 has been previously linked to the NF-κB signaling pathway, including its interaction with the NF-κB transcription factor RelB (Chen et al., 2009; Harman et al., 2019). These findings underscore the intricate interplay among Ehmt2, inflammatory mediators, and the NF-κB signaling pathway, shedding light on the molecular mechanisms underlying inflammatory responses across various disease contexts. In particular, our findings regarding acute pancreatitis offer valuable insights into how dysregulation of epithelial cells can drive inflammatory responses and influence disease progression within the pancreas, while also potentially informing our understanding of related conditions, such as chronic pancreatitis, autoimmune pancreatitis, and pancreatic cancer.

We also report that Ehmt2 functions as a critical regulator of transcriptional programs essential for maintaining pancreatic acinar cell function. These cells must cyclically produce large amounts of enzymes for macromolecule digestion, necessitating tight coordination of nuclear functions, RNA and ribosome production in the nucleolus, mRNA splicing in the spliceosome, protein synthesis in abundant rough ER, and cytoplasmic ribonucleoprotein-rich granules for storage (Logsdon and Ji, 2013). Conditional *Ehmt2* inactivation in acinar cells leads to dysregulation of genes influencing the way the entire organ reacts to inflammation. This dysregulation occurs via two major cellular mechanisms: increased activity of gene networks antagonizing the cell cycle and alterations in this transcriptional regulator that modify expression of molecular mediators, exacerbating acute pancreatitis severity. Furthermore, our findings suggest that *Ehmt2* inactivation also affects nuclear architecture and chromatin organization, potentially influencing gene cluster regulation implicated in pancreatitis pathogenesis. For instance, *Ehmt2* inactivation leads to derepressing the Beta Globin LCR, a regulatory region known for its involvement in internal chromosome looping (Deng et al., 2012). This potentially reflects reorganization of the 3D nucleus, suggesting a broader role for Ehmt2 beyond direct transcriptional regulation and potentially influencing higher-order chromatin structures and nuclear organization. Emphasizing the interconnected nature of epigenetic regulation, transcriptional control, and nuclear organization in shaping inflammatory responses, future investigations into this phenomenon promise to offer further valuable insights into the molecular mechanisms underlying pancreatitis development.

In light of recent findings highlighting the enduring epigenetic memory of inflammatory injury in pancreatic acinar cells (Falvo et al., 2023), the current study’s investigations into *Ehmt2* knockout in this specific cell population during acute pancreatitis gains significance. At the core of our study lies a gene regulatory mechanism within pancreatic acinar cells, revealing how epigenetic dysregulation within this single cell type can instigate a cascade of events, ultimately leading to an amplified injury-inflammation-repair response across the entire organ. This discovery underscores the interconnectedness of cellular processes, where alterations in gene regulation within one cell type propagate across the organ’s cellular landscape, influencing its overall function and response to stimuli. Understanding these systemic effects will provide critical insights into organ homeostasis and disease progression. Moreover, shedding light on the interplay between Ehmt2-mediated cell-specific epigenetic regulation and its broader effects on organ-level responses, our research provides a foundation for targeted interventions aimed at modulating Ehmt2-related epigenetic memory and alleviating the long-term consequences of pancreatic injury.

Limited studies in acute pancreatitis have highlighted the involvement of epigenetic mechanisms, such as histone acetylation and methylation, as well as the therapeutic potential of targeting bromodomain and extra-terminal (BET) proteins and histone deacetylases (HDACs) in mitigating inflammation and reducing disease severity (Bombardo et al., 2017). Building upon this foundation, the insights gained from our study hold promise for the identification of Ehmt2 as another potential therapeutic target, given its pivotal role in modulating inflammatory responses within the pancreas, thus adding to the growing list of epigenomic targets for intervention in pancreatic diseases. Strategies aimed at restoring Ehmt2 function or inhibiting its downstream effectors could offer avenues for mitigating pancreatic inflammation and improving disease outcomes. Furthermore, the identification of Ehmt2’s regulatory network provides a foundation for exploring combinatorial therapies that target multiple nodes within the inflammatory cascade. Such approaches may offer synergistic effects and enhance therapeutic efficacy. In the face of increasing global incidence and complications of acute pancreatitis, our study opens new avenues for therapeutic exploration in pancreatic diseases by elucidating the role of epigenetic regulators and their potential as targets for intervention.

In conclusion, our experiments in two complementary mouse models reveal the multifaceted role of Ehmt2 in regulating gene expression and inflammation during acute pancreatitis, exacerbating pancreatic inflammation upon inactivation. This advances our understanding of Ehmt2’s role in maintaining homeostasis and preventing severe inflammation. Importantly, these findings have implications beyond pancreatology, extending to inflammatory conditions in other organs, as recently evidenced by involvement of Ehmt2 in autoimmune pathways, such as those observed in the colonic mucosa (Ramos et al., 2023). Additionally, the application of spatial transcriptomics further strengthens our results. Considering the ongoing evaluation and testing of Ehmt2 inhibitors in preclinical studies in Sickle Cell Anemia, which also affect Beta Globin genes, and combination therapies for pancreatic cancer, our findings warrant careful consideration regarding their potential to attenuate pancreatic defenses against inflammatory stimuli (Yuan et al., 2013; Krivega et al., 2015; Renneville et al., 2015; Pan et al., 2016; Katayama et al., 2020; Urrutia et al., 2020; Takase et al., 2023). Overall, our study not only presents novel insights but also holds significant biomedical relevance in the context of autoimmune diseases, chronic inflammation, and emerging therapeutic strategies involving Ehmt2.

## Data Availability

The data presented in the study are deposited in the Gene Expression Omnibus repository, accession numbers GSE269251 and GSE269256.
