# Supplementary material for: Ehmt2 inactivation in pancreatic epithelial cells shapes the transcriptional landscape and inflammation response of the whole pancreas
Source: Front Genet. 2024 Jun 14;15:1412767. doi: 10.3389/fgene.2024.1412767 (PMC11211573; doi:10.3389/fgene.2024.1412767)
Supplement: Supplementary file 1 [file DataSheet1.PDF]

Supplementary Material

for

EHMT2 Inactivation in Pancreatic Epithelial Cells Shapes the Transcriptional Landscape and Inflammation Response of the Whole Pancreas

Gareth Pollin[1,2], Angela J. Mathison[1,2], Thiago M. de Assuncao[1,2], Anju Thomas[1,2], Atefeh Zeighami [1], Ann Salmonson[2], Hongfei Liu[1], Guillermo Urrutia[2], Pallavi Vankayala[1], Stephen J Pandol[4], Johnny C. Hong[5], Michael T. Zimmermann[1,3,6], Juan Iovanna[7], Victor X. Jin[1,8], Raul Urrutia*[1,2,3], and Gwen Lomberk*[1,2,9]

Supplementary Figure 1

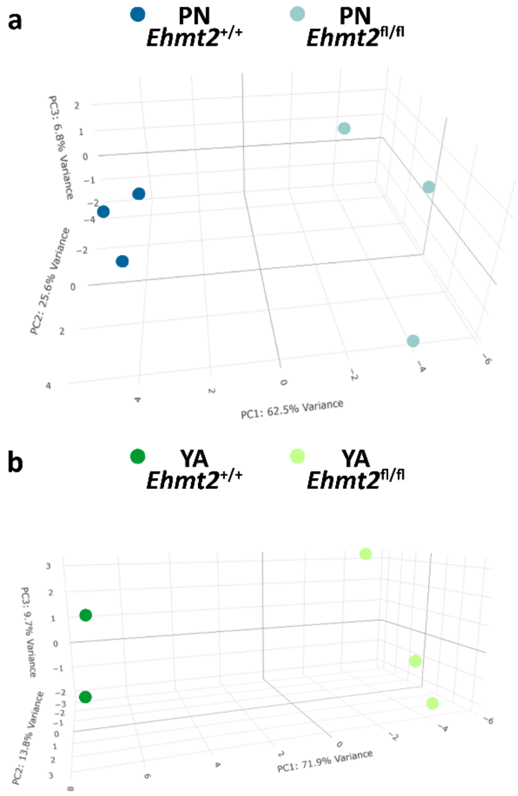

Figure S1. *Ehmt2* inactivation modulates shows a defined separation from WT animals. PCAs are shown based on DEGs from RNA-seq conducted on pancreas tissue from (**a**) *Pdx1-Cre;EHMT*$^{+/+}$ (n = 3) and *Pdx1-Cre;EHMT2*$^{fl/fl}$ (n = 3) mice at Postnatal (PN) day 10 and (**b**) *Pdx1-Cre;EHMT*$^{+/+}$ (n = 2) and *Pdx1-Cre;EHMT2*$^{fl/fl}$ (n = 3) young adult (YA) mice at 4-5 weeks.

Supplementary Figure 2

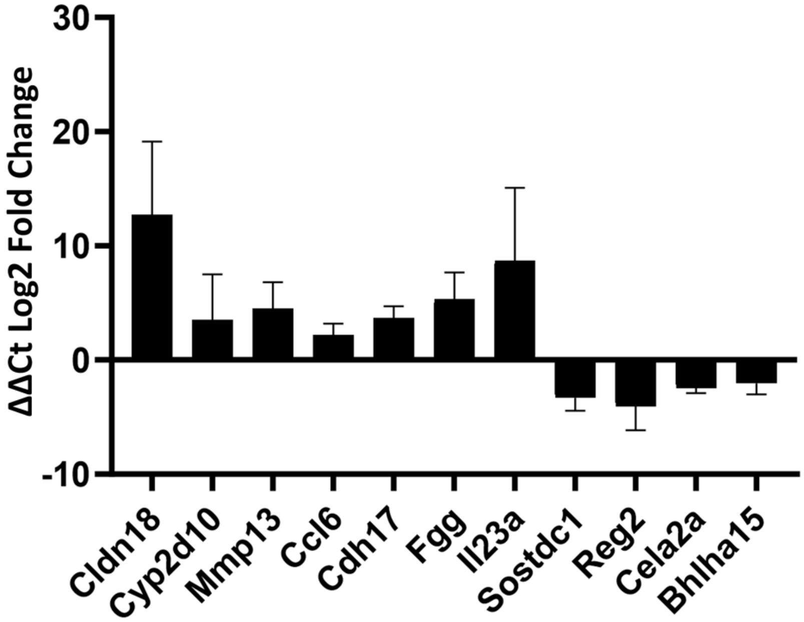

Figure S2. RT-qPCR validation of DEGs during acute pancreatitis with *Ehmt2* inactivation. 7 gene targets that were upregulated and 4 genes that were downregulated during acute pancreatitis in *Ehmt2*$^{fl/fl}$ animals with acute pancreatitis compared to *Ehmt2*$^{+/+}$ animals were selected among the most significant genes from the RNA-seq dataset to confirm changes in transcript levels. *Hprt* was used as a housekeeping control to normalize across samples. Bar graph depicts ΔΔ CT Log2 FC of *Ehmt2fl/fl* vs *Ehmt2+/+* animals (n=3) with data expressed as mean ± SEM.

Supplementary Figure 3

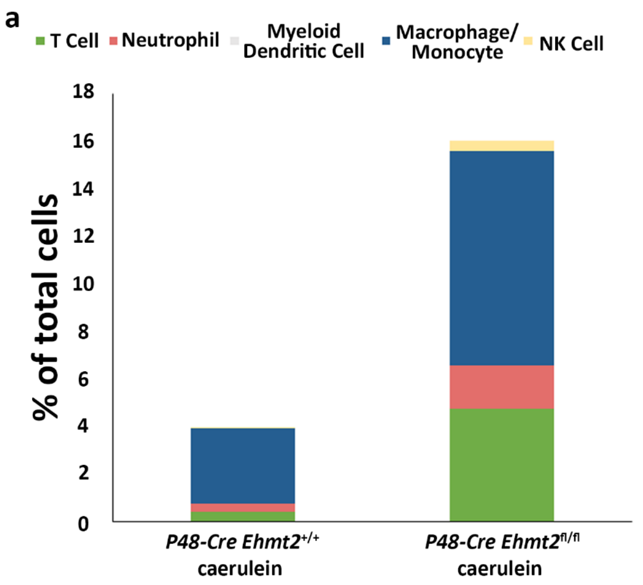

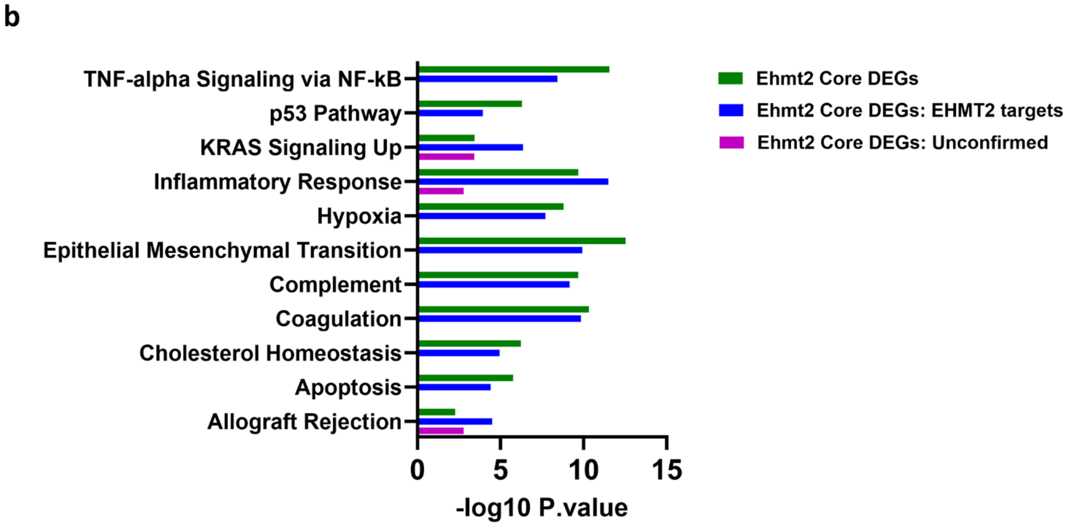

Figure S3. Ehmt2 deficiency driven by *P48-Cre* also promotes immune cell infiltration in injured pancreatic tissue via transcriptomic landscape alterations. (**a**) MCPcounter deconvolution analysis predicts immune cell composition in the total cell population after acute pancreatitis induction with caerulein treatment using bulk RNA-seq data from *Ehmt2*$^{+/+}$ and *Ehmt2*$^{fl/fl}$ animals on the *P48-Cre*$^{+/+}$ background. (**b**) MSigDB Hallmarks pathway enrichment analysis of significant core DEGs from *Pdx1-Ehmt2*$^{fl/fl}$ and *P48-Ehmt2*$^{fl/fl}$ acute pancreatitis models, subdivided into Ehmt2 targets based on the ChIP-Atlas dataset and those unconfirmed as direct Ehmt2 targets. Enrichment data is plotted as -log10 p value.
